# A general ink formulation of 2D crystals for wafer-scale inkjet printing

**DOI:** 10.1126/sciadv.aba5029

**Published:** 2020-08-12

**Authors:** Guohua Hu, Lisong Yang, Zongyin Yang, Yubo Wang, Xinxin Jin, Jie Dai, Qing Wu, Shouhu Liu, Xiaoxi Zhu, Xiaoshan Wang, Tien-Chun Wu, Richard C. T. Howe, Tom Albrow-Owen, Leonard W. T. Ng, Qing Yang, Luigi G. Occhipinti, Robert I. Woodward, Edmund J. R. Kelleher, Zhipei Sun, Xiao Huang, Meng Zhang, Colin D. Bain, Tawfique Hasan

**Affiliations:** 1 Cambridge Graphene Centre, University of Cambridge, Cambridge CB3 0FA, UK.; 2 Department of Electronic Engineering, The Chinese University of Hong Kong, Shatin, Hong Kong.; 3 Department of Chemistry, Durham University, Durham DH1 3LE, UK.; 4 College of Optical Science and Engineering, Zhejiang University, Hangzhou 310027, China.; 5 School of Electronic and Information Engineering, Beihang University, Beijing 100191, China.; 6 Institute of Advanced Materials, Nanjing Tech University, Nanjing 210009, China.; 7 Department of Physics, Imperial College London, London SW7 2AZ, UK.; 8 MQ Photonics, Department of Engineering, Macquarie University, New South Wales, Australia.; 9 Quantum Matter Institute, University of British Columbia, Vancouver, BC V6T 1Z4, Canada.; 10 Department of Electronics and Nanoengineering, QTF Centre of Excellence, Aalto University, Tietotie 3, FI-02150 Espoo, Finland.

## Abstract

Recent advances in inkjet printing of two-dimensional (2D) crystals show great promise for next-generation printed electronics development. Printing nonuniformity, however, results in poor reproducibility in device performance and remains a major impediment to their large-scale manufacturing. At the heart of this challenge lies the coffee-ring effect (CRE), ring-shaped nonuniform deposits formed during postdeposition drying. We present an experimental study of the drying mechanism of a binary solvent ink formulation. We show that Marangoni-enhanced spreading in this formulation inhibits contact line pinning and deforms the droplet shape to naturally suppress the capillary flows that give rise to the CRE. This general formulation supports uniform deposition of 2D crystals and their derivatives, enabling scalable and even wafer-scale device fabrication, moving them closer to industrial-level additive manufacturing.

## Teaser

A binary solvent ink exploiting solutal Marangoni flows to suppress the coffee-ring effect for uniform printing of 2D crystals.

## INTRODUCTION

**Fig. 1:**
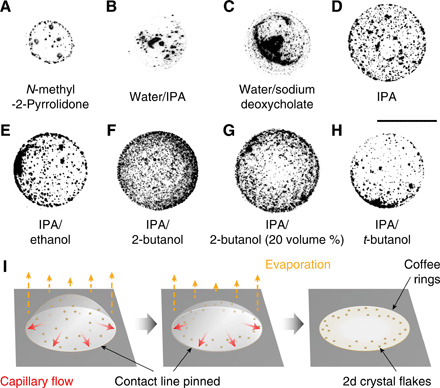
Coffee-ring effect. Inverted optical micrographs of dried inkjet-printed droplets on clean glass: (**A** to **C**) common solution-processed 2D crystal dispersions (sections S1 and S2). (**D** to **H**) Formulated inks via solvent exchange in IPA or binary solvents of IPA/ethanol (10 volume %), IPA/2-butanol (10 and 20 volume %), and IPA/*t*-butanol (10 volume %). Scale bar, 50 μm. The brightness and contrast are optimized for clarity. MoS_2_ is the 2D crystal example. Substrate is Si/SiO_2_ at 60°C. (**I**) Schematic drying process showing CRE formation (*8*).

The wide spectrum of distinct and yet complementary properties of two-dimensional (2D) crystals offers huge potentials for (opto)electronics, photonics, and sensor development (*1*, *2*). Engineering the 2D crystals also allows the fabrication of their hybrids and heterostructures, with an even more diverse set of properties for a substantially expanded application scope. In recent years, remarkable efforts have been devoted to adapting 2D crystals to functional printing toward their scalable and low-cost device fabrication (*3*). For this, the most common approach is to exfoliate their bulk crystals through chemical or ultrasonic assisted processes into mono- and few-layer flakes. These stably suspended dispersions are then directly used for device fabrication, showing glimpses of their exciting potential in recent advances (*4*–*6*). However, this direct adaption without elaborate ink formulation through control over composition, rheology, and fluidic properties presents challenges in achieving uniformly deposited functional structures and, hence, device reproducibility and scalability. The three critical parameters behind this are suboptimal droplet jetting (section S2), poor control over substrate wetting (section S2), and drying of the inks (Fig. 1, A to C). Although various approaches have been proposed to realize stable jetting and appropriate wetting (section S2), a strategy to suppress nonuniform deposition during drying of the deposited droplet, the coffee-ring effect (CRE), remains elusive.

The CRE in a drying droplet requires two essential conditions (Fig. 1I) (*7*, *8*): first, that the contact line is pinned and, second, that the droplet adopts a spherical-cap shape to minimize its surface-free energy. Simple geometric considerations then result in radially outward capillary flow to replenish solvent evaporating near the contact line. Note that capillary flow refers to Laplace pressure–driven flows of liquid arising from variations in the curvature of the liquid-air interface. Faster solvent evaporation at the contact line than at the apex enhances this capillary flow, which carries dispersed solutes to the contact line and deposits them there, leading to a ring-shaped stain. Because of this fundamental drying mechanism, suppression of the CRE is currently a major challenge in ink formulations of 2D crystals. Although various strategies have been developed to suppress the CRE (*9*, *10*), none of these are generally applicable for 2D crystal inks due to problems of dispersion stability, postprocessing requirements, or the effect of ink additives on material functionality. In particular, CRE-induced nonuniform deposition for additive-free 2D crystal inks is considerable on uncoated and nonporous substrates such as polyethylene terephthalate (PET) and Si/SiO_2_.

Our previous discovery of uniform deposits of black phosphorus surmised that Marangoni effects, the flows caused by surface tension gradients, might be a determining factor to suppress the CRE in mixed alcohol inks (*11*). However, a detailed experimental understanding of the drying mechanism of these inks, key to the applicability of this approach to the wider 2D material family, was lacking. Here, with new experimental investigation, we present, to our knowledge, a new mechanism of natural suppression of the CRE and rationalize the exquisite sensitivity to the choice of the alcohols used (Fig. 1, D to H). We propose a general design principle for ink formulation to print uniform patterns of 2D crystals and their derivatives, enabling scalable manufacturing of planar functional devices such as gas sensors and photoconductors with highly reproducible properties.

## RESULTS

We investigate isopropanol (IPA)–based alcohol mixtures in our ink formulation. IPA is widely used as a solvent for graphics inks and, recently, for 2D crystal inks (*3*). It supports a metastable dispersion of the 2D crystal nanoflakes, and its low surface tension ensures good wetting of substrates (*3*). However, as we observe, ring stains persist with IPA-based 2D crystal inks (Fig. 1D). Binary mixtures of IPA with ethanol and *t*-butanol also produce nonuniform deposits, but a mixture of IPA with 2-butanol [optimally 10 volume percent (volume %)] suppresses the CRE (Fig. 1, E to H, and section S4). To understand the differing behaviors of the alcohol mixtures, we consider their surface tensions, γ, and evaporation rates (table S1). The blends are all zeotropic and show only small deviations from ideality. During drying, the concentration of the less volatile component is enhanced at the contact line. The radial surface tension gradient, dγ/dr, for IPA/ethanol is <0 (ethanol evaporates faster, enriching the contact line with lower surface tension IPA), >0 for IPA/2-butanol, and ≈0 for IPA/*t*-butanol (similar volatilities).

**Fig. 2:**
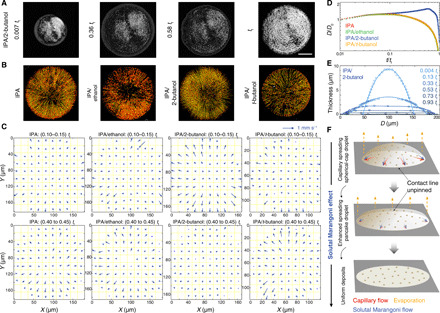
Solutal Marangoni effect. (**A**) Optical micrographs of a drying droplet of IPA/2-butanol (10 volume %) with tracer particles on glass from droplet impact (0.007 *t*_f_), to maximum spreading (0.36 *t*_f_), to when the edge recedes close to the particle periphery (0.58 *t*_f_), and at the end of drying (*t*_f_). Scale bar, 50 μm. The brightness and contrast are optimized for clarity. (**B**) Particle trajectories at 0.04 *t*_f_ to 0.05 *t*_f_, with red arrows showing the trajectory end, and (**C**) the corresponding velocity maps averaged over two time intervals: For IPA, IPA/ethanol, and IPA/*t*-butanol, the particles nearest to the pinned contact lines display largest velocities and increase from ∼0.3 to ∼0.6 mm s^−1^; for IPA/2-butanol, the contact line and the near particle first advance at ∼0.5 mm s^−1^. The droplet then ceases to expand, and the particle velocities greatly decrease. Polystyrene nanoparticles (diameter ~755 nm) are used as tracer particles as 2D crystals are too small to visualize. Stationary particles adhering to substrates are excluded from analysis. (**D**) Contact diameter (*D*) of droplets without tracer particles as a function of time. The time is normalized by *t*_f_ and *D* by the diameter just after (1 ms) inertial droplet impact (*D*_0_). (**E**) Reconstructed height profiles of an IPA/2-butanol droplet at various times (movies S6 and S8). Solid line at 0.004 *t*_f_ and 0.13 *t*_f_ is circular fit. (**F**) Schematic depicting the solutal Marangoni effect.

To visualize the flows arising from these surface tension gradients, we seed IPA and the blend droplets with polystyrene tracer particles (Fig. 2, section S3, and movies S1 to S4). Radially outward flows with no recirculation are observed in all these droplets. Plots of the particle trajectories show that IPA, IPA/ethanol, and IPA/*t*-butanol behave similarly, but IPA/2-butanol is markedly different (Fig. 2, B and C). In the first three cases, the droplets are pinned at the early stage of drying, <0.1 *t*_f_ (where *t*_f_ is the drying time); the particle velocities are greatest when close to the contact line and increase during drying. The IPA/2-butanol droplet, however, continues to expand until 0.36 *t*_f_ with particles near the contact line moving at the same speed as the contact line; after the droplet ceases to expand, the particle velocities reduce greatly.

Tracer particles perturb flows when the height of the droplet approaches the diameter of the particles (*12*). Therefore, we also study the spreading and drying behavior of these four solvent systems without the tracer particles (Fig. 2D). In all the cases, the droplets dissipate their kinetic energy ≈ 1 ms after impact (*13*) and spread in diameter during 0.01 *t*_f_ to 0.03 *t*_f_ following a power law, *D* ≈ *t*^n^ with *n* ≈ 0.14, slightly larger than the value of 0.1 expected from Tanner’s Law when the gravitational force is negligible due to droplet size (*14*). IPA, IPA/ethanol, and IPA/*t*-butanol then show a long and slow retraction of the contact line, following *D* ∝ (*t*_f_ − *t*)*^n^* with *n* = 0.52 ± 0.01 during drying phase of 0.68 *t*_f_ to 0.97 *t*_f_ (section S3 and fig. S4), close to the value of 0.5 expected for diffusion-limited evaporation with a constant contact angle (*15*). These three fluids follow theoretical “universal curves” for evaporation of a pure wetting fluid in the absence of thermal Marangoni effects (*16*). This agreement does not prove that thermal Marangoni effects are not present but shows that they do not noticeably influence the drying dynamics. The consonance of the three curves demonstrates further that solutal Marangoni effects do not influence the shape evolution in the IPA/ethanol or IPA/*t*-butanol mixtures.

In lubrication theory (*17*), the evolution equation for the droplet height, *h*, depends on a dimensionless parameter, *C =* ε^3^*/*3*Ca*, where the capillary number *Ca =* η*u/*γ (where ε is the aspect ratio *h*_0_*/R*_0_, η is the viscosity, *u* is the characteristic radial velocity, and γ is the surface tension). *C* represents the relative importance of height changes due to flows caused by differences in Laplace pressure (capillary flows) and those due to evaporation. When *C* ≫ 1, capillary flows readily balance the loss of liquid by evaporation, and the droplet shape remains close to a spherical-cap. Conversely, when *C* ≪ 1, capillary flows are negligible, and the height evolution is determined by the local evaporation rate (in the absence of Marangoni effects). For these three cases, we find *C* = 10 at the beginning of spreading [where we have taken *h*_0_ and *R*_0_ to be the height and radius immediately after impact (*t* ~ 1 ms; ε ~ 0.18) and *u* to be the contact line speed of 2.4 mm s^−1^], *C* = 2 at maximum spreading, and *C* ~ 1 during the retraction phase. Although *C* is not always ≫1, side-view measurements of the droplet profile at early times and interferometric profiles at later times show that the droplet profile is always well-fitted by a spherical-cap (section S3 and fig. S4). In the presence of particles that pin the contact line, capillary flows are expected to lead to ring stains (*7*, *8*), which is what we observe (Fig. 1).

The IPA/2-butanol mixture shows remarkably different dynamics after the initial spreading phase (*t* < 0.05 *t*_f_). The droplet continues to spread for about 60% of the drying time, at an almost constant speed, followed by a sharp transition into rapid contact line retraction. The retraction does not follow a power law (fig. S4). Reconstructed profiles show that IPA/2-butanol changes from the initial spherical-cap into a flattened pancake shape as early as 0.33 *t*_f_, which persists through the remainder of drying (Fig. 2E). Immediately after impact, the value of *C* is similar to that of the other three solutions; the very different spreading behavior shows that there must be additional terms in the height evolution equation. Once the droplet adopts a pancake shape, the curvature is small, resulting in an absence of Laplace pressure gradients to drive radial flow (except in close proximity to the contact lines). Consequently, the droplet thins uniformly as it evaporates, leading to a uniform deposit. The dominance of evaporation over capillary flows can also be inferred from a calculation of *C* for the pancake shaped droplets; if we use droplet properties half-way through drying, we find *C* = 10^−2^, which is << 1.

We propose that CRE suppression in IPA/2-butanol arises from solutal Marangoni-enhanced spreading (Fig. 2F) (*18*–*20*). After the initial capillary-driven spreading (*t* < 0.05 *t*_f_; Fig. 2D), spreading is strongly coupled with drying until 0.6 *t*_f_. Faster evaporation of IPA enriches the contact line with 2-butanol, leading to a surface tension gradient acting from the droplet apex to the contact line. This shear stress accelerates the radially outward spreading flow, as shown in the velocity map in Fig. 2C. The surface tension gradient can be estimated from the velocity gradient: We estimate the difference in the surface tension between the apex and contact line as O(10^−5^ Nm^−1^), only 1% of the surface tension difference between the two solvents (section S3). In Marangoni-driven spreading, the velocity at the free surface is twice the mean of radial velocity. The nanoparticle-enriched zone at the (moving) contact line is constantly overtaken by fresh solution from the center, maintaining a uniform concentration profile. The persistence of the pancake shape during drying can be understood qualitatively as follows. For small Peclet numbers, the composition of the droplet is uniform with height. The rate of change of composition with evaporation is inversely proportional to the thickness of the film. Consequently, thicker areas of the drop are richer in IPA and, therefore, have a lower surface tension than thinner areas, leading to a Marangoni flow from thicker areas to thinner areas. We propose that this natural negative feedback mechanism assists the suppression of the CRE. We note that the thermo-Marangoni effect on suppression of the CRE is negligible (*21*) and has been discussed in section S3.

**Fig. 3:**
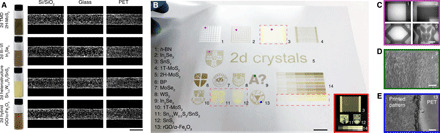
Inkjet printing of 2D crystals. (**A**) Ink examples and corresponding optical micrographs of printed single lines on Si/SiO_2_. Scale bar, 100 μm. The inks are diluted for clarity. Brightness and contrast of the lines are enhanced to reflect the distribution of printed flakes. TMD, transition metal dichalcogenide; *h*-BN, hexagonal boron nitride. (**B**) Printed patterns on PET: (1 to 13) individual patterns and (14) gradient printing. Scale bar, 1 cm. Inset shows 3, 11, 12, and 14 with a dark background. Corresponding (**C**) optical micrographs of selected areas in 1, 2, 3, and 5. Scale bar, 500 μm. (**D** and **E**) SEM in 9 and 13. Scale bars, 100 μm. Photo credit: Guohua Hu, University of Cambridge and The Chinese University of Hong Kong.

With this mechanistic understanding, we synthesize >10 different 2D crystals, their heterostructures (solution grown 2D-2D material systems) and hybrids (solution grown 0D-2D material systems), and formulate their inks (section S4 and fig. S5). We present inkjet printing of these inks in Fig. 3. Uniform flake distributions are achieved across all the printed single lines on the substrates (Fig. 3A), underscoring the ability of our inks to suppress the CRE. The optical absorbance measured from the printed patterns linearly scales with the print repetition (errors <2.5%; fig. S9). The pattern-to-pattern variation is also minimal (errors <5%; fig. S10), underscoring highly controlled and uniform printing achievable with our inks. The printing process can be scaled up for large-area patterning (Fig. 3B). Figure 3C presents the corresponding high-resolution optical micrographs of selected areas, demonstrating that the patterns are uniform without CRE. Close-up examination through scanning electron microscopy (SEM) also confirms this (Fig. 3, D and E). In particular, Fig. 3E shows sharp, clear edges of the prints in spite of prolonged print sessions involving 60 print repetitions. The ink formulation is applicable for other material systems, for example, nanoparticles (e.g., polystyrene nanobeads) and organic salts (e.g., oxalates; fig. S6). We propose the formulation may also be suitable for ink pigment particles in a similar size range if stable suspension can be achieved. The inks can also be spray coated for highly uniform coating (section S5 and fig. S7).

With regard to the previous printed 2D crystal device demonstrations, there have been two primary obstacles for real-world applications. First, the current solution-based synthesis or processing of 2D crystals limits their properties compared to those produced via alternative methods. This is primarily due to the smaller and irregular flake dimension inherent to solution processing techniques. This typically translates to inferior performance metrics in devices such as transistors or photodetectors, compared to those produced from mechanical exfoliation or high-temperature synthesis of 2D crystals. The second challenge is the consistency in the device performance as a result of nonuniform printing of 2D crystals, largely due to the CRE. Printed planar devices leveraging the properties of solution-processed 2D crystals, such as gas sensors, hold enormous application potential if such performance variabilities are addressed. This demands considerable innovations in the underlying ink formulation strategies. Our work focuses on the solvent carrier design. In this work, we exploit our ink formulation to address the second challenge and investigate the device-to-device variabilities of three types of inkjet-printed 2D crystal devices. However, we note that the CRE may not necessarily be independent of the dimension of the 2D crystal nanoflakes and that enhanced control of the dimensions may be exploited to refine their interparticle capillary interactions for additional CRE suppression (*22*).

**Fig. 4:**
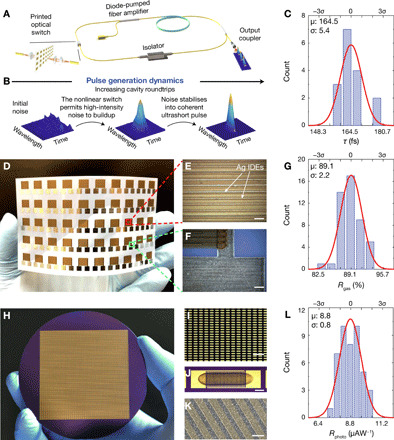
Inkjet-printed patterns with identical print-to-print functional features. (**A**) Schematic of ultrafast fiber laser cavity showing integration of an array of 4 × 8 inkjet-printed 2H-MoS_2_ SAs. (**B**) Spectrogram plots illustrating the evolution of ultrashort laser pulse generation. (**C**) Gaussian fitting of the measured pulse duration τ of randomly selected 16 SA devices. (**D** to **F**) Fully inkjet-printed rGO/α-Fe_2_O_3_ 5 × 10 sensor array on PET. Scale bars, 100 μm. Photo credit: Guohua Hu, University of Cambridge and The Chinese University of Hong Kong. (**G**) Gaussian fitting of the measured responsivity (*R*_gas_, the change in the device resistance) of at 1 part per million NO_2_. Three short-circuited devices are excluded from fitting. (**H**) Photograph of 100 × 45 inkjet-printed MoS_2_ photodetector array on a 3-inch wafer (diameter 76 mm), where the uniformly printed 2H-MoS_2_ over interdigitated gold electrodes acts as the active layer. Photo credit: Yubo Wang, University of Cambridge and Zhejiang University. (**I**) Photograph of >300 devices, showing visually identical printed MoS_2_ array. Scale bar, 2 mm. (**J**) Optical micrograph of a single photodetector. Scale bar, 100 μm. (**K**) SEM micrograph showing even flake distribution over the false-colored interdigitated electrodes. Scale bar, 10 μm. (**L**) Gaussian fitting of the measured responsivity *R*_photo_ of a randomly selected 50-device array under 40-μW illumination at 635 nm, 5 V. Six devices are short-circuited and excluded from the fitting.

As demonstrations, here, we print arrays of nonlinear optical saturable absorbers (SAs; 4 × 8 array), room temperature gas sensors (5 × 10 array), and photodetectors (45 × 100 array) with 2H-MoS_2_ and 2D hybrid rGO/α-Fe_2_O_3_ (Fig. 4). These 2D crystals, especially in their solution-processed forms, are widely used in these applications (*1*, *23*, *24*). Assessment of 16 randomly selected SAs shows uniform duration of the ultrafast pulses generated, fitting a Gaussian distribution with 3.3% spread in SD (σ) from the mean (μ; Fig. 4C). Likewise, the printed room temperature NO_2_ sensors and photodetectors show a respective spread of 2.5% (Fig. 4G) and 9.1% in their response (Fig. 4L). See sections S7 to S9 for further details. These narrow spreads are substantially lower than what can be achieved with the solution-processed N-methyl-2-pyrrolidone (NMP)–based dispersions (fig. S14). In comparison, depending on the complexity of the circuit, a variation of ±10 to ±30% in threshold voltage in printed thin-film transistors is considered acceptable for passive radio frequency sensors in an industrially scalable printing process (*25*). Our reported narrow spreads in performance are therefore well within acceptable statistical variations for printed device manufacturing from 2D crystal inks, addressing one of the most challenging obstacles toward their additive manufacturability.

## DISCUSSION

Through experimental observations, we have introduced a new drying mechanism that uses Marangoni-enhanced spreading to suppress the CRE through contact-line unpinning and droplet deformation. Exploitation of this formulation for inkjet printing of 2D crystals enables scalable device fabrication, with highly consistent and reproducible properties. Further understanding of this mechanism could allow adaptation of solvent mixtures beyond those investigated here, considerably expanding its applicability for the wide range of 2D crystals and other material platforms, including nanoparticles and organics. Reliable printing of such a wide range of optically and electrically active materials and their mixtures will substantially boost the fabrication of complex emerging devices. Our ink formulation with active CRE suppression thus lays the foundation for high-speed additive manufacturing of all-printed sensors and systems with a high level of large-scale integration.

## MATERIALS AND METHODS

### Solution processing methods

The 2D crystal flakes, including graphene, transition metal dichalcogenides [molybdenum disulfide (MoS_2_), molybdenum diselenide (MoSe_2_), and tungsten disulfide (WS_2_)], hexagonal boron nitride (*h*-BN), bismuth telluride (Bi_2_Te_3_), indium selenide (In_2_Se_3_), and black phosphorus (BP), are produced via previously reported liquid-phase exfoliation, ion intercalation, and chemical synthesis. The 2D crystal heterostructure (solution-phase epitaxy grown Sn_0.5_W_0.5_S_2_ nanoplates onto SnS_2_ nanoplates, Sn_0.5_W_0.5_S_2_/SnS_2_) is produced via solution-based epitaxy. The graphene hybrid (reduced graphene oxide with spindle-like α-Fe_2_O_3_, rGO/α-Fe_2_O_3_) is synthesized via hydrothermal method. See detailed solution processing methods and the material characterizations in section S1.

### Droplet-drying study

Details of the setups and the analysis of the radial flows of sessile droplets and their spreading and drying are available in section S3.

### Ink formulation and characterization

The produced 2D crystals are redispersed in mixtures of IPA and alcohol (anhydrous alcohols for BP) through 10-min sonication for ink formulation. The ink concentration is adjusted to 1 g liter^−1^. The 1 T-MoS_2_ ink contains 9 volume % water to stabilize the flakes. Polystyrene nanobead solution (10 weight %; Sigma-Aldrich) is diluted with IPA/2-butanol (10 volume %) by 50 times for ink formulation. *N*,*N*,*N*′,*N*′-tetramethyl-4,4′-diaminotriphenylcarbenium oxalate (Sigma-Aldrich) is dissolved in IPA/2-butanol (10 volume %) at a concentration of 5 g liter^−1^ for ink formulation. The typical surface tension, viscosity, and density of the inks are 28 mNm^−1^, 2 mPas, and 0.8 gcm^−3^, respectively, giving an inverse Ohnesorge number of 10. This ensures stable jetting of individual droplet corresponding to each electrical drive pulse. Commercial silver ink (Sigma-Aldrich) is used as received for printed Ag interdigitated electrodes (IDEs) for the gas sensors.

### Inkjet printing and characterization

Fujifilm Dimatix Materials Printer DMP-2831 is used. The ink cartridge is Dimatix DMC-11610, with a jetting nozzle diameter of 22 μm. The volume of individual droplets generated is 10 pL. For a typical printing process, the substrates [Si/SiO_2_ (oxide thickness of 100 nm), glass, and PET (thickness of 50 μm)] are cleaned with acetone/IPA/deionized water before printing. For printing of the SAs, a 1.5-μm-thick PET is used as provided. For ease of handing, this PET is laminated onto a 100-μm-thick PET support before printing; it can be easily peeled off for device integration after printing. For printing of the sensors, a PET substrate with porous coating (Mitsubishi) is used as received. To prevent buildup of agglomeration of 2D crystals around the nozzles, cleaning is conducted via purging the nozzles with an IPA/2-butanol mixture before and after each print repetition. For SEM characterization of inkjet-printed 2D crystals, a ~6-nm-thick gold layer is sputtered before imaging.

## Supplementary Material

aba5029_Movie_S8.mp4

aba5029_Movie_S6.mp4

aba5029_Movie_S1.mp4

aba5029_Movie_S2.mp4

aba5029_Movie_S5.mp4

aba5029_SM.pdf

aba5029_Movie_S7.mp4

aba5029_Movie_S4.mp4

aba5029_Movie_S3.mp4

## SUPPLEMENTARY MATERIALS

Supplementary material for this article is available at http://advances.sciencemag.org/cgi/content/full/6/33/eaba5029/DC1
