# Supplementary material for: A general ink formulation of 2D crystals for wafer-scale inkjet printing
Source: Sci Adv. 2020 Aug 12;6(33):eaba5029. doi: 10.1126/sciadv.aba5029 (PMC7423364; doi:10.1126/sciadv.aba5029)
Supplement: aba5029_SM.pdf [file aba5029_SM.pdf]

Science Advances
AAAS

advances.sciencemag.org/cgi/content/full/6/33/eaba5029/DC1

Supplementary Materials for

A general ink formulation of 2D crystals for wafer-scale inkjet printing

Guohua Hu, Lisong Yang, Zongyin Yang, Yubo Wang, Xinxin Jin, Jie Dai, Qing Wu, Shouhu Liu, Xiaoxi Zhu, Xiaoshan Wang, Tien-Chun Wu, Richard C. T. Howe, Tom Albrow-Owen, Leonard W. T. Ng, Qing Yang, Luigi G. Occhipinti, Robert I. Woodward, Edmund J. R. Kelleher, Zhipei Sun, Xiao Huang, Meng Zhang*, Colin D. Bain*, Tawfique Hasan*

*Corresponding author. Email: mengzhang10@buaa.edu.cn (M.Z.); c.d.bain@durham.ac.uk (C.D.B.); th270@cam.ac.uk (T.H.)

Published 12 August 2020, *Sci. Adv.* **6**, eaba5029 (2020)
DOI: 10.1126/sciadv.aba5029

The PDF file includes:

Sections S1 to S9
Figs. S1 to S15
Table S1

Other Supplementary Material for this manuscript includes the following:

(available at advances.sciencemag.org/cgi/content/full/6/33/eaba5029/DC1)

Movies S1 to S8

Section S1. Production of 2d crystals via solution processing

Liquid-phase exfoliation (LPE) and ion intercalation: We use LPE to produce 2d crystals of graphene, transition metal dichalcogenides (TMDs: molybdenum disulfide (MoS_2), molybdenum diselenide ($MoSe_2$), and tungsten disulfide (WS_2)), hexagonal boron nitride (*h*-BN), bismuth telluride (Bi_2Te_3), indium selenide (In_2Se_3) and black phosphorus (BP). To ensure exfoliation and stable dispersions, LPE requires the use of high boiling point solvents (e.g. N-Methyl-2-pyrrolidone (NMP)) that have matched Hansen solubility parameters (*25*, *26*), or solvent mixtures (e.g. water/isopropyl alcohol (IPA)) with minimized Hansen solubility parameter distance (*26*), or surfactants (e.g. sodium deoxycholate (SDC) in water) for electrostatic and/or steric stabilization (*27*).

For LPE, 100 mg bulk crystals (10 mg for BP) are mixed with 10 mL NMP (anhydrous NMP for BP), water/IPA (55 vol.%/45 vol.%) or water/SDC (SDC concentration 7 gL^{-1}). The bulk graphite, TMDs, *h*-BN, Bi_2Te_3 and In_2Se_3 are purchased from Sigma. The bulk BP is purchased from Smart-Elements. The mixtures are sonicated in a 20 kHz bath sonicator for 12 hours at 15°C, and then centrifuged at 4,000 rpm for 1 hour. The upper 70% of the resultant dispersions are collected for inkjet printing and spray coating without further ink formulation following previous reports, as well as ink formulation through solvent exchange (see Section S4).

As will be discussed in Section S4, certain 2d crystals (for instance *h*-BN and In_2Se_3) produced by this method may not formulate dispersions with sufficient stability for inkjet printing. Therefore, in addition to LPE, we also use ion intercalation assisted exfoliation to produce *h*-BN, In_2Se_3 and MoS_2, following the methods reported in Refs. (*28*, *29*). For this, 1 g bulk crystals of MoS_2, In_2Se_3 and *h*-BN are mixed into 30 mL of water and 1 mL hydrazine hydrate (Sigma), followed by 30 min bath sonication. The mixtures are then transferred into autoclaves and heated at 120°C for 8 hours. The treated MoS_2, *h*-BN and In_2Se_3 are washed and dried, and then mixed into 3 mL 1.6 M butyllithium solution in hexane (Sigma-Aldrich) for 48 hours. The resultant MoS_2, *h*-BN and In_2Se_3 are washed with hexane (60 mL) and dispersed in water via 1 hour bath sonication. The exfoliated MoS_2, *h*-BN and In_2Se_3 are next washed with water for three times. The washed MoS_2 is dispersed in 20 mL water and sonicated for 1 hour, followed by 1 hour centrifugation at 4,000 rpm. The upper 70% of the resultant dispersion is collected for ink formulation (see Section S4). To differentiate between the MoS_2 produced via LPE and ion intercalation, they are named as 2H-MoS_2 and 1T-MoS_2, respectively (*28*, *29*). The washed *h*-BN and In_2Se_3 are dispersed in 20 mL water/IPA (55 vol.%/45 vol.%) and sonicated for 1 hour, followed by 30 min centrifugation at 1,000 rpm. The upper 70% of the resultant dispersions are collected for ink formulation (see Section S4).

Chemical synthesis: The 2d crystals of tin disulfide (SnS_2) and $Sn_{0.5}W_{0.5}S_2/SnS_2$ heterostructure are produced via chemical synthesis, following the method reported in Ref. (*30*). In a typical synthesis process of SnS_2, 0.25 mmol $SnCl_4 \cdot 5H_2O$ (Sigma) and 3.75 mmol $CS(NH_2)_2$ (J&K chemical, Shanghai) are dissolved in 19.45 mL water and stirred for 2 hours. This solution is then transferred to an autoclave, and heated at 220°C for 12 hours. The obtained product is then centrifuged at 8,000 rpm for 10 min. The obtained precipitate is washed with water for three times for ink formulation (see Section S4). In a typical synthesis process of $Sn_{0.5}W_{0.5}S_2/SnS_2$, 0.25 mmol $(NH_4)_{10}H_2(W_2O_7)_6$, 7.5 mmol $CS(NH_2)_2$, and 0.5-0.625 mmol $SnCl_4 \cdot 5H_2O$ are dissolved in 19.45 mL water and stirred at 80°C for 2 hours. This solution is then transferred to an autoclave, and heated at 220°C for 60 hours. The obtained product is then centrifuged at 8,000 rpm for 10 min. The precipitate is then washed with water for three times for ink formulation (see Section S4). We

show in Fig. S2a-d SEM and TEM micrographs of the SnS_2 and $Sn_{0.5}W_{0.5}S_2/SnS_2$ samples. In particular, Fig. S2c,d show that the $Sn_{0.5}W_{0.5}S_2$ nanoplates have been successfully grown on SnS_2.

Hydrothermal synthesis: The graphene hybrid, i.e. reduced graphene oxide decorated with spindle-like α-Fe_2O_3 (rGO/α-Fe_2O_3) is produced via hydrothermal synthesis. In a typical process, 5.6 mg graphene oxide monolayer flakes (99% of the flakes are monolayers; Tanyuanhuigu, Shanghai) are dispersed in 11.25 mL water, followed by 30 min bath sonication. The GO dispersion is mixed with an $FeCl_3$ (Sigma) solution (36.5 mg in 9 mL water), and added with another 13.5 mL IPA and 180 mg CH_3COONa. The resultant mixture is sonicated for 30 min, transferred to an autoclave and heated at 120°C for 8 hours. The produced rGO/α-Fe_2O_3 is washed with water for three times for ink formulation (see Section S4).

Material characterizations: We characterize the thickness and lateral dimension of the produced 2d crystals used in inkjet printing demonstrations; Fig. S1. These include 2H-MoS_2, WS_2, $MoSe_2$ and BP produced via LPE, 1T-MoS_2, *h*-BN and In_2Se_3 produced via ion intercalation, $Sn_{0.5}W_{0.5}S_2/SnS_2$ and SnS_2 produced via chemical synthesis, and rGO/α-Fe_2O_3 produced via hydrothermal synthesis. The thickness and lateral dimension are typically acquired by AFM, except the thickness histograms of the $Sn_{0.5}W_{0.5}S_2/SnS_2$ and SnS_2 that are acquired through SEM. Since the SnS_2, $Sn_{0.5}W_{0.5}S_2/SnS_2$ and rGO/α-Fe_2O_3 are not produced via typical top-down exfoliation techniques, they are also characterized with SEM and TEM; Fig. S2. The AFM samples are characterized with a Bruker Dimension Icon AFM in ScanAsyst^{TM} mode, using a silicon cantilever with a silicon nitride tip. The SEM samples are characterized with JEOL JSM-7800F. The TEM samples are characterized with JEOL 2100Plus.

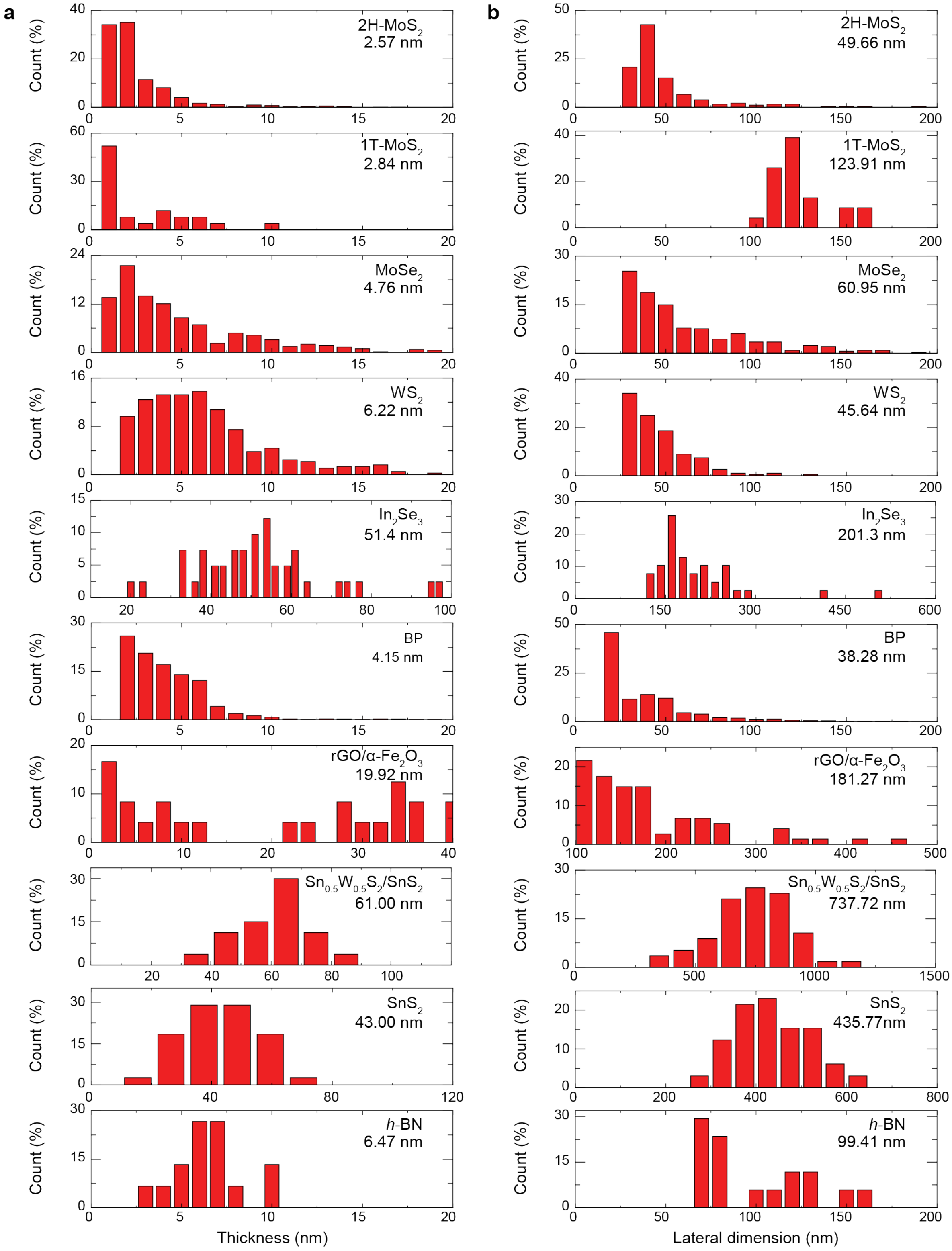

Fig. S1. Characterization of the produced 2d crystals. Distribution of (a) thickness and (b) lateral dimension of the 2d crystals, with the average values presented.

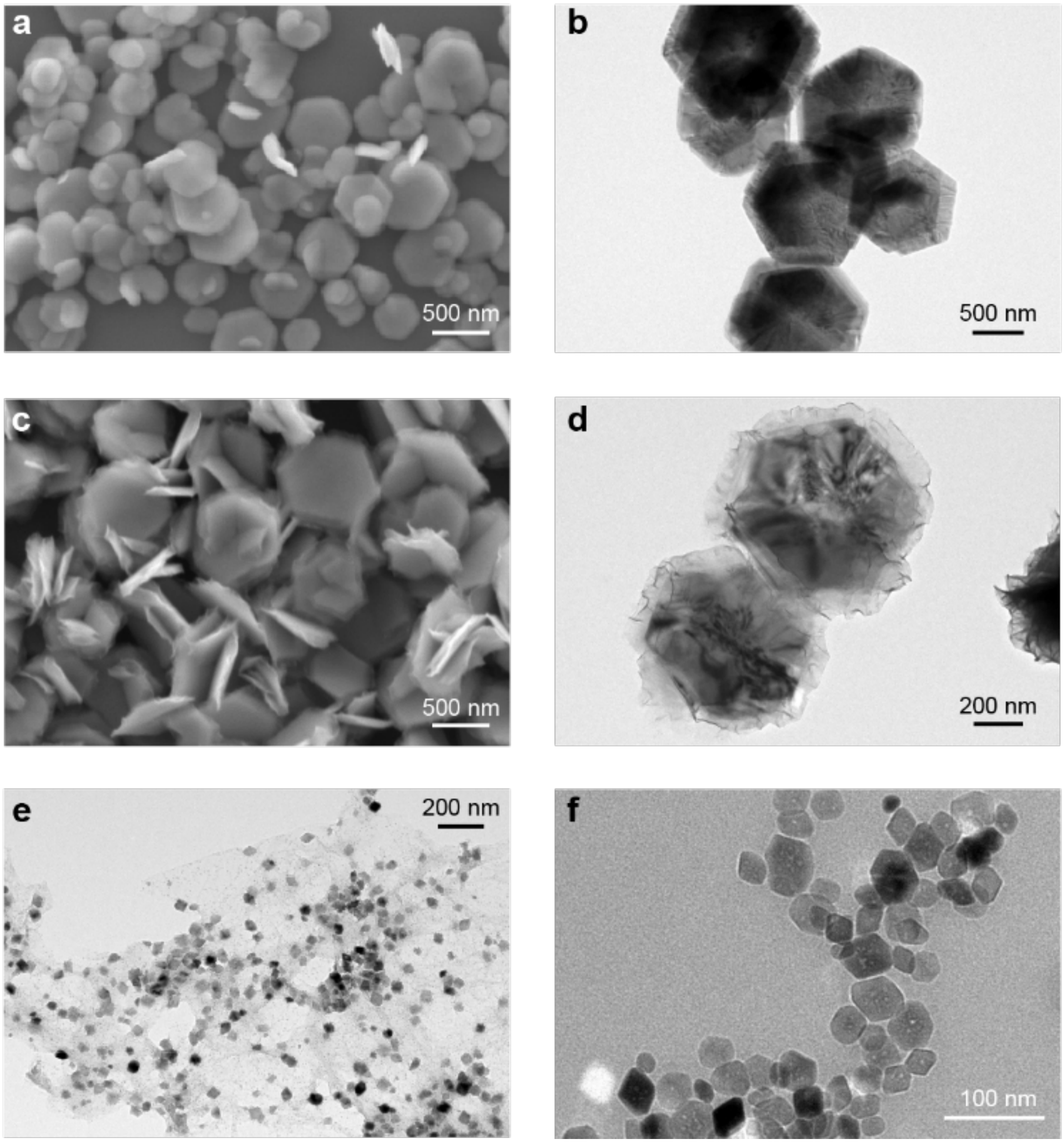

Fig. S2. Characterization of the synthesized 2d crystals. (a) SEM and (b) TEM micrographs of typical SnS_2 2d crystals; (c) SEM and (d) TEM micrographs of typical $Sn_{0.5}W_{0.5}S_2/SnS_2$ flakes, showing $Sn_{0.5}W_{0.5}S_2$ nanoplates are grown on the SnS_2 flakes. (e,f) TEM micrographs of rGO/α-Fe_2O_3, showing the α-Fe_2O_3 nanoparticles are grown on the rGO flakes.

Section S2. Inkjet printing and spray coating of the solution-processed dispersions

LPE dispersions of 2d crystals have been commonly adapted to inkjet printing without further ink formulations (3–6). These include the dispersions in organic solvents (e.g. NMP), solvent mixtures (e.g. water/IPA), and water with surfactants (e.g. water/SDC) (*3*, *31*).

In Table S1, we show the inverse *Ohnesorge* numbers (*Z*) of these LPE dispersions and the common solvents used. Generally, the Z value of an ink is used to determine whether it would form a stable jetting of individual droplets under each electrical drive pulse: $Z = \sqrt{\gamma\rho a}/\eta$, depending on the viscosity (η), surface tension (γ), and density (ρ), and the inkjet cartridge nozzle diameter (a) (*4*). An established guiding principle is that Z should be 1-14 to avoid the formation of satellite droplets (>14) or elongated ligaments (<1) during the jetting process.

As shown in Table S1, Z values of the NMP and water/SDC based LPE dispersions are >15, while the value for water/IPA based dispersions approach 14. This indicates that these LPE dispersions tend to generate satellite droplets (*4*, *32*). Indeed, jetting of such satellite droplets is observed with NMP based dispersions; Fig. S3a. This can even lead to jetting deviations from the jetting path, as shown in Fig. S3b, causing the droplets to deposit on untargeted areas. As a result, printing using these dispersions can lead to nonuniformity, as demonstrated in Fig. S3c,d where the typical morphologies with one single and multiple print repetitions on Si/SiO_2 are presented. In addition to unstable jetting, heavy coffee rings are also formed with these dispersions at the edges of each of these single and overlapped droplets.

Meanwhile, as shown in Fig. S3c,d, inkjet printing with these LPE dispersions also cannot appropriately wet the Si/SiO_2 substrate for a continuous feature with either single or multiple print repetitions. Under such wetting conditions, the droplets would nonuniformly retract during drying, resulting in poor uniformity. To allow appropriate wetting for a consistent deposit, the ink surface tension should be 7-10 mNm^{-1} lower than the substrate surface energy; otherwise, the deposited droplets retract and ball-up during drying (*33*). With such high surface tensions of >32 mNm^{-1}, these dispersions therefore cannot wet the substrates commonly used in electronics and optoelectronics, including Si/SiO_2 and glass (36 mNm^{-1}) (*34*) and plastics such as PET (48 mNm^{-1}) (*35*). A commonly adopted solution to enhance wetting is to increase the substrate surface energy via surface treatments, e.g. by oxygen plasma. Alternatively, lowering the surface tension of the inks by adding e.g. low-surface tension solvents such as alcohols or additive surfactants and polymers is also viable and widely adopted. However, the use of additives could be detrimental as they disrupt formation of an electrically continuous network between individual nanostructures. In this work, we focus on additive-free ink formulation using a mixed alcohol carrier, with a surface tension of <30 mNm^{-1} for wetting of a wide range of substrates without the need for surface treatments. We note that spray coating with these NMP, water/SDC and water/IPA based dispersions on PET also fails to develop a continuous deposited feature; Fig. S3e,f. In this case, 0.5 mL dispersions are sprayed at room temperature.

As shown in Table S1, alcohols (e.g. IPA and the butanols) tend to give Z values within the 1-14 range, suggesting that these alcohols are suitable solvents for inkjet ink formulation. Indeed, alcohols such as IPA have been widely used in graphics (*36*) and recent 2d crystal ink formulations (*3*, *31*). Also, their low surface tensions (<30 mNm^{-1}) tend to ensure adequate wetting of the substrates. However, as demonstrated in Fig. 1d and as will be seen in Movie S5, pure alcohols do not suppress the CRE, and leads to nonuniform deposits.

Table S1. Physical properties and the Z values for some common solvents and the 2d crystal dispersions and the formulated inks. The temperature is 20°C unless otherwise stated. The vapor pressure (P_V) is calculated by Antoine equation ($\lg P_V = A + B/T + C\lg T + DT + ET^2$), where T is the absolute temperature in K and P_V is in mmHg in the equation and converted to kPa in the table. The Antoine parameters, A, B, C, D and E are taken from Ref. (*37*). Pendant droplet measurement and parallel plate rheometer are used to measure the surface tension and the viscosity of the LPE dispersions and the formulated inks, respectively. The nozzle diameter of the Dimatix DMC-11610 cartridge, a, is 22 μm.

	P_V (kPa)	Z parameters			$Z = \frac{\sqrt{\gamma\rho a}}{\eta}$
		γ (mNm^{-1})	η (mPas)	ρ (gcm^{-3})	
**Common solvents**					
NMP		40.7 (*38*)	1.7 (*38*)	1.0 (*38*)	18
Water		72.9 (*39*)	1.0 (*40*)	1.0 (*41*)	40
Ethanol	5.8	22.4 (*39*)	1.2 (*38*)	0.8 (*41*)	17
IPA	4.4	21.3 (*39*)	2.4 (*38*)	0.8 (*38*)	8
2-butanol	1.7	22.9 (*39*)	3.8 (*38*)	0.8 (*38*)	5
t-butanol	4.0	20.7 (*42*)	4.3 (25°C) (*43*)	0.8 (25°C) (*43*)	4
**LPE dispersions**					
NMP based		42	2.0	1	15
Water/IPA based		32	2.0	0.9	13
Water/SDC based		45	0.8	1	39
**IPA/2-butanol (10 vol.%) inks**		28	2.2	0.8	10

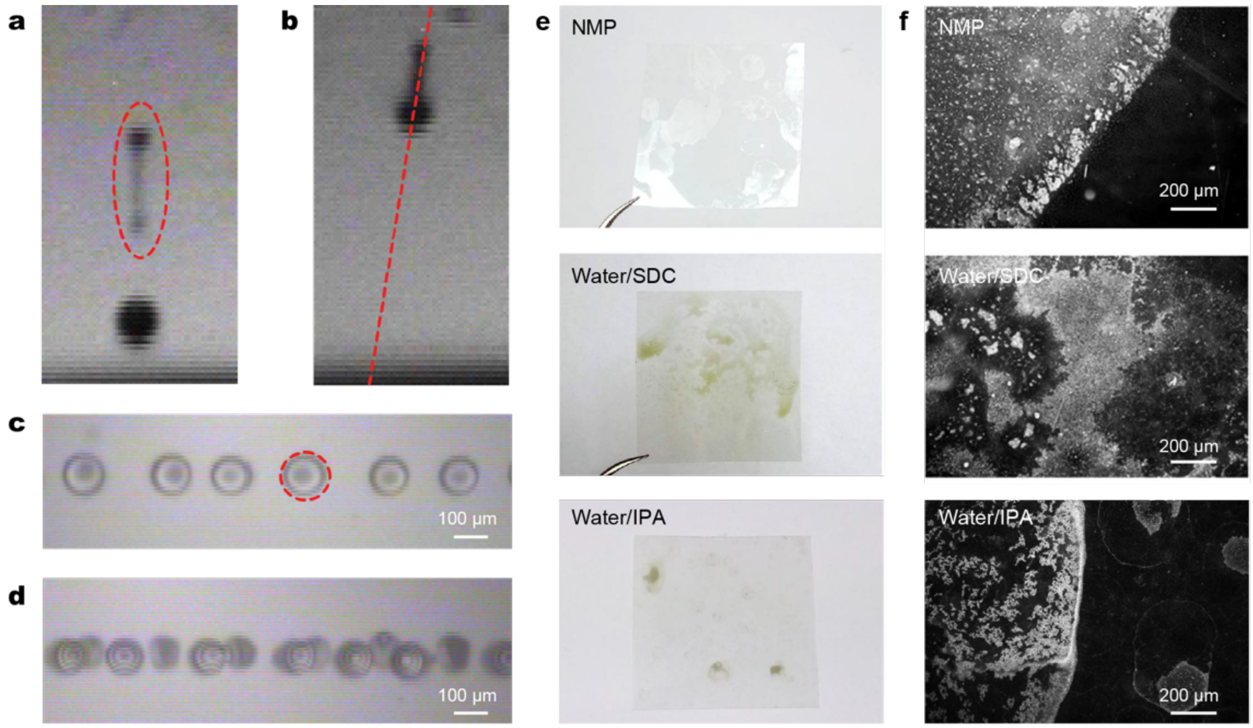

Fig. S3. Inkjet printing with the solution-processed dispersions. Typical micrographs for the generation of (a) satellite droplet as indicated by the dashed circle, and (b) jetting deviation from the intended vertical patch, as indicated by the dashed line, observed through the inkjet printer stroboscopic camera. Typical micrographs for printing morphologies with (c) one single print repetition and (d) multiple print repetitions on Si/SiO_2, showing coffee rings and poor-wetting; the red circle in (c) indicates a coffee-ring. (e) Photographs of sprayed films (3 × 3 cm) on PET and (f) corresponding optical dark-field micrographs. Demonstrated with 2H-MoS_2 LPE dispersions. Photo Credit: Guohua Hu, University of Cambridge and The Chinese University of Hong Kong.

Section S3. Solutal Marangoni effect of the mixed solvent ink formulation

Supplementary Movies

Drying sessile droplets with tracer particles

Movie S1: The flows of a drying pL-droplet from IPA with the tracer particles by dark-field imaging. Drop drying time $t_f = 178$ ms.

Movie S2: The flows of a drying pL-droplet from IPA/ethanol (10 vol.%) with the tracer particles by dark-field imaging. Drop drying time $t_f = 201$ ms.

Movie S3: The flows of a drying pL-droplet from IPA/2-butanol (10 vol.%) with the tracer particles by dark-field imaging. Drop drying time $t_f = 138$ ms.

Movie S4: The flows of a drying pL-droplet from IPA/*t*-butanol (10 vol.%) with the tracer particles by dark-field imaging. Drop drying time $t_f = 125$ ms.

Movie S5: The flows of a drying pL-droplet from 2-butanol with the tracer particles by dark-field imaging. Drop drying time $t_f = 444$ ms. This is a controlled experiment to demonstrate that 2-butanol cannot suppress the CRE.

Drying sessile droplets without tracer particles

Movie S6: Spreading and drying of a pL-droplet from IPA/2-butanol (10 vol.%) by film interferometry. Drop drying time $t_f = 148$ ms. The Movie is used for droplet profile reconstruction in Fig. 2e at 0.13 t_f, 0.33 t_f, 0.53 t_f, 0.73 t_f and 0.93 t_f.

Movie S7: Spreading and drying of a pL-droplet from IPA/ethanol (10 vol.%) by film interferometry. Drop drying time $t_f = 150$ ms.

Movie S8: Spreading and drying of a pL-droplet from IPA/2-butanol (10 vol.%) by shadow imaging in synchronization with Movie S6. The Movie is used for droplet profile reconstruction in Fig. 2e at 0.004 t_f.

Note that the respective drying time for the IPA and IPA/*t*-butanol (10 vol.%) droplets without tracer particles is 188 ms and 174 ms.

Scale bars in the movies (Supplementary Movie S1-S8) are 40 μm.

Materials

The solvents used in the experiments are as follows: IPA (Fisher scientific, Laboratory reagent grade), ethanol (Fisher scientific, analytical regent), 2-butanol (Sigma-Aldrich, Reagent 99%), and *t*-butanol (Alfa Aesar, ACS reagent grade 99%+). PVP- stabilized [poly(N-vinylpyrrolidone)] PS (polystyrene) particles (755 nm) (preparation in Ref. (*44*)) are used as the tracer particles in all experiments. Dry PVP-PS particles are weighed to make suspension in a range of 0.04-0.06 wt.% through 30 min bath sonication. The suspensions exhibit no obvious sedimentation over a day. Microscopic glass coverslip (Academy Science, 0.13-0.17 mm thickness) is cleaned by acetone/ ultrapure water/IPA/N_2 stream.

Experimental methods

Droplets of a typical volume of 40-50 pL are dispensed onto the glass coverslip from a drop-on-demand device (MJ-ABP-01, MicroFab Technologies, nozzle diameter 30 μm) under a bipolar waveform through a MicroFab device (JetDrive III controller CT-M3-02). The set-ups for the

study of the flows of a drying droplet and the film formation have been presented elsewhere (*45*, *46*). In brief, in the flow setup, the sample is illuminated by a light-emitting diode (505 nm, Thorlabs) from an angle so that only scattered light from particles can be collected by the objective lens (50x/0.6, Nikon) underneath the substrate and hence, dark-field image is obtained. The flow is recorded by a high-speed camera (APX-RS, Photron). For film interferometric imaging, another light-emitting diode light (505 nm, Thorlabs) is used to illuminate the sample underneath the substrate. The reflected light from the interfaces of liquid-air and liquid-solid interferes and fringes can be observed when the thickness of the film is less than the coherent length of the light source, where the fringe contrast is also limited by reflectance of two interfaces of the droplet. Two neighboring bright or dark fringes have a thickness difference of $\lambda/(2n')$, where λ is the light wavelength and n' is the refractive index of the liquid sample, i.e. approximately 180 nm in our experimental condition. Both the image views can be switched on at the same time though a separate view that gives a better image contrast. A shadow view from the side of the sessile droplet is also utilized for the determination of initial droplet volume and the contact angle at the early stage of spreading via an objective lens (20x/0.42, Edmund Optics) and a high-speed camera (CR450x3, Optronis). Cameras and MicroFab controller are synchronized from the same trigger. The imaging system is calibrated by a micro calibration plate (Lavision), giving a resolution of 0.35 μm/pixel for the view from underneath and 0.58 μm/pixel from side, respectively. All the experiments are carried out at room temperature of 21 ± 1°C and relative humidity of 45 ± 5% (note that in moist air, water may condense into the drying alcohol droplets and affect their spreading dynamics). The particles are traced, and the speed is calculated over time and region binning with a custom-written Matlab code adapted from Ref. (*47*). The contact line of the spreading film is traced and analyzed as a function of time, and the droplet shape is reconstructed from interference fringes when droplet spreads and thins to a thickness of less than 3 μm.

Discussion of thermal and solutal Marangoni effect

Typically, compositional surface tension gradients are orders of magnitude larger than the thermal ones at ambient conditions (*21*). Chen *et al.* recently investigated the Marangoni effects on the drying of a pure solvent and binary solvent mixtures on a hydrophobic substrate with a heat control from 22-80°C (*48*). They showed that for a pure water droplet the thermal Marangoni effect appears only when the substrate is heated to >50°C. The pure 1-butanol droplet (less volatile than water) does not show any thermal instability at any heated temperature. However, 5% 1-butanol/water mixture shows solutal Marangoni effect at any substrate temperate. Jambon-Puillet *et al.* also studied very recently the spreading dynamics of sessile droplets of alkenes with the vapor pressure varied over two orders of magnitude (from nonane of 0.59 kPa to pentane of 53 kPa) (*49*). They showed a good agreement in droplet spreading for heptane (vapor pressure of 4.6 kPa) and the other less volatile alkenes where the thermal effect is neglected in their spreading model. This again infers that the thermal effect is trivial for medium volatile solvents.

We have shown in the manuscript that IPA, IPA/ethanol and IPA/*t*-butanol droplets without tracer particles have almost identical behavior that falls onto theoretical 'universal curves' for the case of complete wetting of a pure fluid in the absence of thermal Marangoni effects (*16*). Their spreading curves at (0.01-0.03) t_f (Fig. 2d) are fitted with power law exponents close to the predicted values under a simple model of sessile droplet subject to capillary and viscous forces. Here we replot Fig. 2d as Fig. S4a. In their evaporation phase during (0.68-0.97) t_f, a fit in $D \approx (1-t/t_f)^n$ gives us n as 0.52 for IPA, 0.53 for IPA/ethanol, and 0.51 for IPA/*t*-butanol. These slopes lie between the value of 0.50 (the 'D^2 law') predicted from a diffusive model with a constant contact

angle (*15*) and the value of 0.54 predicted in a recent analysis by Saxton *et al*. (*50*). These exponents are slightly higher than the values obtained from microliter drops of pure alkanes reported in the range of 0.42-0.49 (*51*, *52*). The results suggest that there is no obvious sign of thermal Marangoni flows in these three cases and that coffee rings are formed in a similar manner.

On the contrary, IPA/2-butanol has enhanced spreading (Fig. 2d), where the mixed solvent droplet spreads outwards for 60% of the drying time, preventing the formation of a deposit at the contact line. We note that the enhanced spreading also occurs in the experiments described above where the droplet contains tracer particles. The presence of these tracer particles reduces the duration of the spreading phase from 0.6 t_f (without particles) to 0.36 t_f (with tracer particles). The spreading and drying are strongly coupled and leads to a complex drying curve (Fig. S4a) with a simple power law fit only at final stage of drying (0.89-0.97) t_f, where we get an exponent of 0.62.

We have shown in Fig. 2e that the droplet shape of IPA/2-butanol without tracer particles is transformed dramatically from a spherical-cap shape to a 'pancake' shape as early as 0.33 t_f. The internal flow reduces speed greatly around (0.40-0.45) t_f (Fig. 2b,c). Note that the droplet height profile at 0.004 t_f is reconstructed from side-view shadow image, while the others are reconstructed interferometric profiles. For dynamics, refer to Movie S6 and S8. In Fig. S4b, we compare interference microscopy images of IPA/ethanol and IPA/2-butanol droplets without tracer particles around halfway through drying (0.45 t_f). As shown, the IPA/ethanol droplet is an almost perfect spherical-cap despite the presence of a Marangoni stress along the free surface acting from the contact line towards the apex: capillarity dominates the shape. In contrast, the IPA/2-butanol droplet (which is still spreading outwards at 0.45 t_f) has a 'pancake' profile with an approximately uniform thickness of 1 μm over the contact diameter of ~200 μm before curving to meet the substrate with a contact angle of ~3°.

We attribute the unique spreading and evaporation behavior of IPA/2-butanol to a solutal Marangoni effect. The surface tension difference needed to drive this flow in the IPA/2-butanol can be estimated from the increase in radial velocity of particles at early times (when the droplet shapes are the same) compared to the pure IPA (Fig. 2b,c). As we compare the flow velocities between IPA/2-butanol and IPA at (0.1-0.15) t_f, the particle speed is almost doubled in the former case. We attribute the extra value of ~0.2 mm s^{-1} (near the contact line) to be Marangoni stress induced. The tangential component of viscous stress is balanced by the stress associated with surface tension gradients, i.e. $d\gamma = \eta(u/h)R \sim 10^{-5}$ Nm^{-1}, where γ is the surface tension, η is the viscosity, u is the Marangoni flow speed of 0.2 mm s^{-1}, h is the droplet height of ~1 μm at 1/3 of the drying time, and R is the contact radius of 80 μm. That is to say that the surface tension difference between the contact line and the apex is O(10^{-5} Nm^{-1}) compared to a surface tension difference between the two pure solvents of O(10^{-3} Nm^{-1}).

The average evaporation rate of a sessile droplet from a pure fluid can be estimated by (*53*):

$$-\dot{m} \approx \pi R D_V \rho_V (0.27\theta^2 + 1.30) \approx 4 R D_V \rho_V \quad (1)$$

where R is the droplet contact radius, D_V is the diffusion coefficient of the vapor, $\rho_V = M_W P_V/(R_{gasconstant}\, T)$ is the vapor density via the ideal gas law, $R_{gasconstant} = 8.3$ Jmol^{-1}K^{-1} is the gas constant, T is absolute temperature in K, M_W is the molecular weight, and P_V is the vapor pressure. Note θ is ≤1 in our pinned case. Our droplet initial volume is 40-50 pL (say 45 pL) and R is 80 μm for the pinned cases. The diffusion coefficient is 1.18 ×, 0.993 ×, 0.880 × and 0.873 × 10^{-5} m^{2}s^{-1} for ethanol, IPA, 2-butanol and *t*-butanol, respectively (*54*). The average evaporation rate is 4.1 ×, 3.4 ×, 1.4 × and 3.4 × 10^{-10} kgs^{-1} for ethanol, IPA, 2-butanol and *t*-butanol, respectively.

IPA therefore evaporates about 2.4 times as fast as 2-butnaol and will be depleted first at the contact line as the vapor flux is the highest at the contact line. The enhanced spreading in IPA/2-butanol strongly indicates that the preferential evaporation of IPA (the lower surface tension component) has created a surface tension gradient at the droplet surface. This Marangoni stress leads a flow established from the droplet apex to the contact line. The nanoparticle-enriched zone at the (moving) contact line is constantly overtaken by fresh solution from the centre, maintaining a uniform concentration profile. IPA and *t*-butanol have almost the same evaporation rates. It is therefore understandable that their mixture will behave like pure IPA. Ethanol evaporates slightly faster than IPA, which may lead to Marangoni flows opposite to the outward capillary flow. However, the suppression of spreading or acceleration of the contact line retraction has not been observed. This indicates that the flows are still capillarity dominated and the coffee-ring deposit therefore forms similarly as IPA and IPA/*t*-butanol. The surface tension of the 90% IPA / 10% co-alcohol mixtures is close to that of IPA – for an ideal mixture it will be $\sigma_{IPA} + 0.1\ \Delta\sigma$, where $\Delta\sigma$ is the difference in surface tensions between the pure co-alcohol and IPA. For IPA/ethanol, the ethanol evaporates faster than the IPA so the surface tension near the contact line moves towards the value for pure IPA. The maximum difference in the surface tension between the apex and the contact line is therefore $0.1\ \Delta\sigma$. For IPA/2-butanol, the IPA evaporates faster and so the liquid near the contact line is enriched in 2-butanol. The maximum surface tension at the contact line is $\sigma_{2\text{-butanol}}$. Hence the maximum surface tension difference between the contact line and the apex is $0.9\ \Delta\sigma$. Consequently, much larger Marangoni stresses can arise in the 9:1 IPA/2-butanol mixture than in the 9:1 IPA/ethanol mixture.

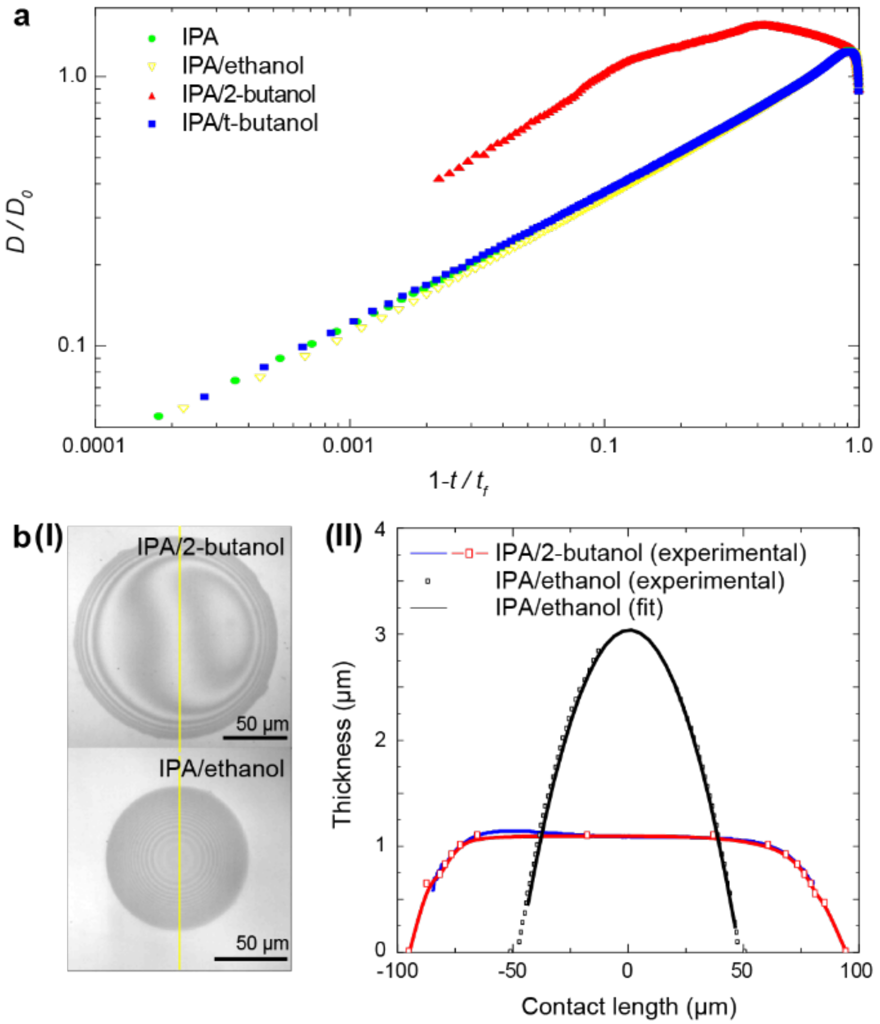

Fig. S4. Contact diameter of spreading droplets and droplet shape profiles. (a) Normalized contact diameter of a drying droplet as a function of normalized remaining drying time for IPA and the IPA-based alcohol binaries. Replotted from Fig. 2d. (b) Interference studies of drying IPA/2-butanol and IPA/ethanol droplets without tracer particles at t = 0.45 t_f: (I) interference micrographs; (II) the reconstructed droplet shape profiles, open squares with *B-spline* line is from cross section of the frame, and blue line is reconstructed from tracing frames near the center of the drop; for IPA/ethanol, open squares is from cross section of the frame, and black line is circle fit. For dynamics, refer to Movie S6 and S7.

Section S4. Formulation and printing of mixed solvent inks

IPA/2-butanol (10 vol.%) inks:

The 2d crystal flakes in the LPE dispersions are extracted through vacuum filtration. These 2d crystals include graphene, BP, *h*-BN, TMDs (2H-MoS_2, $MoSe_2$, WS_2), Bi_2Te_3, and In_2Se_3. Note the flakes extracted from water/SDC are repeatedly washed with water to remove the residual SDC. For ink formulation, these obtained 2d crystals are dispersed in IPA/2-butanol (10 vol.%) through 10 min bath sonication. These inks differ in stability against sedimentation: the graphene, *h*-BN, In_2Se_3 and Bi_2Te_3 inks remain stable for a few hours, insufficient for long-term inkjet printing processes. However, the TMD inks and the BP ink are stable without visible aggregations for up to weeks, sufficient for large-scale inkjet printing.

Since 1T-MoS_2 requires water to remain stable for long-term printing, the obtained water-based 1T-MoS_2 dispersion is diluted with IPA/2-butanol (10 vol.%) by 10 times for ink formulation. The *h*-BN and In_2Se_3 produced via ion intercalation are extracted by vacuum filtration, and then redispersed in IPA/2-butanol (10 vol.%) through 10 min bath sonication for ink formulation.

The washed SnS_2, $Sn_{0.5}W_{0.5}S_2/SnS_2$ and rGO/α-Fe_2O_3 are redispersed in IPA/2-butanol (10 vol.%) through 10 min bath sonication for ink formulation.

Ink formulation strategy:

We start ink formulation from pure IPA. Short-chain alcohols, such as IPA, are widely used in graphics inks (*36*) and recently, in 2d crystal inkjet printable inks (*3*). They present appropriate fluidic properties for satellite-free inkjet printing (Table S1). The low surface tension of IPA also ensures good wetting of high-energy substrates. However, as shown in Fig. 1d, the contact line of pure IPA based inks still pins and CRE persists.

Having demonstrated the limitation with pure IPA, we investigate binary solvent systems, with IPA as the primary solvent. To induce variable Marangoni effects, we choose mixtures of IPA with additional 10 vol.% of ethanol, 2-butanol and *t*-butanol (secondary solvents) for ink formulation. These alcohol combinations are all zeotropic and show only small deviations from ideality. We have listed the surface tensions (γ) and vapour pressures of the alcohols in Table S1. As we have discussed in the manuscript, we expect that the radial surface tension gradient, $d\gamma/dr$, of IPA/ethanol to be <0; >0 for IPA/2-butanol; ≈ 0 for IPA/t-butanol. Figure 1f shows that only IPA/2-butanol suppresses the coffee-ring effect, yielding an even distribution of flakes across the deposit. However, the other mixtures show ring stains. We then conduct further careful investigation on the Marangoni effect in the IPA/2-butanol inks, as presented in the Manuscript.

To experimentally find an appropriate solvent volume ratio for the IPA/2-butanol formulation, we investigate other volume percentages of 2-butanol. Fig. 1g shows the optical micrograph of IPA/2-butanol (20 vol.%). Similar to the 10 vol.% case, the 20 vol.% droplet does not show significant coffee rings, suggesting the coffee-ring effect is also suppressed in this case. However, as we can observe, the 20 vol% droplet shows fuzzier edges and more condensed materials at the edges, as compared to the 10 vol.% droplet. Its diameter is also increased to ~74 μm (~68 μm for 10 vol.%). An increased diameter is not ideal for functional printing as it means a decreased printing resolution in device fabrication. Meanwhile, we observe the 20 vol.% droplet dries slower as more 2-butanol takes a longer time to evaporate. We note that a larger volume (>20 vol.%) of 2-butanol leads to a weaker suppression of the coffee-ring effect, fuzzier edges, larger diameter and also a longer drying time. Based on the above considerations, we chose 10 vol.% for the ink formulation.

We note that besides the IPA/2-butanol (10 vol.%) ink formulation, other alcohol mixtures fitting this solutal Marangoni mechanism are also proven viable, for instance IPA/1-propanol (10 vol.%) and IPA/1-butanol (10 vol.%).

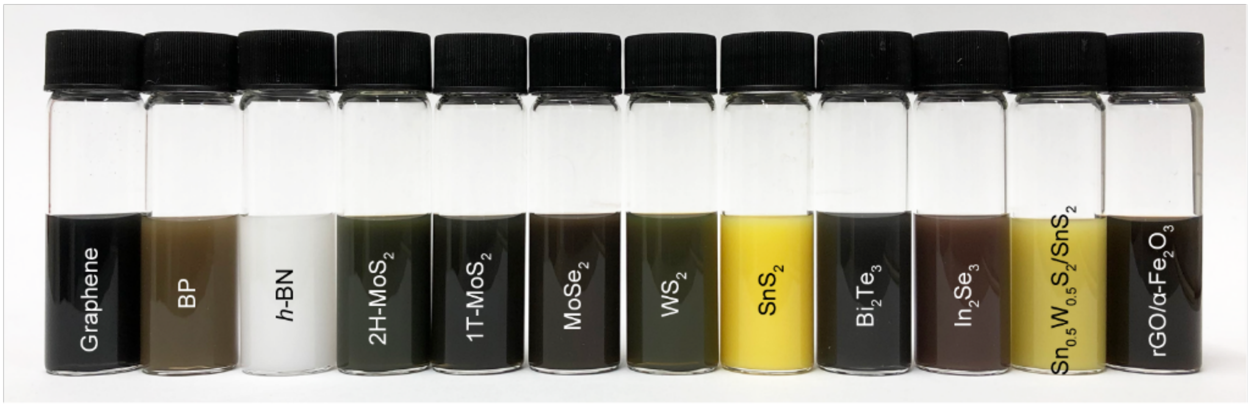

Fig. S5. Photograph of the formulated inks in this work. This includes graphene, BP, TMDs (2H-MoS_2, $MoSe_2$, WS_2) and Bi_2Te_3 produced via LPE, *h*-BN, 1T-MoS_2 and In_2Se_3 produced via ion intercalation, SnS_2 and $Sn_{0.5}W_{0.5}S_2/SnS_2$ produced via chemical synthesis, and rGO/α-Fe_2O_3 produced via hydrothermal synthesis. Typical ink concentration is ~1 gL^{-1}. Photo Credit: Guohua Hu, University of Cambridge and The Chinese University of Hong Kong.

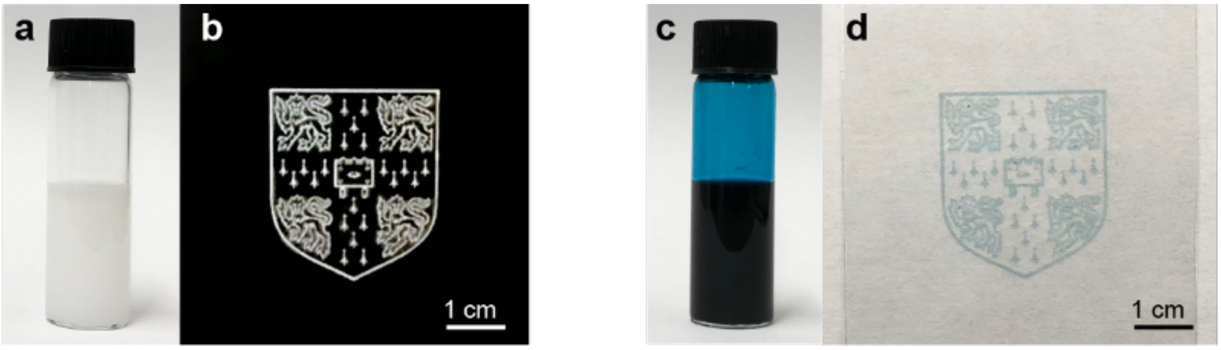

Fig. S6. Inkjet printing of nanoparticle and organic material inks. Inkjet printing of (a,b) nanoparticles and (c,d) organics on PET using the ink formulation of IPA/2-butanol (10 vol.%) solvent composition. Polystyrene nanobeads are used as the nanoparticle example. For ink formulation, polystyrene nanobeads solution (800 nm, 10 wt.%; Sigma) is diluted with IPA/2-butanol (10 vol.%) by 50 times. N,N,N',N'-Tetramethyl-4,4'-diaminotriphenylcarbenium oxalate (Sigma) is used as the organic material example. For ink formulation, it is dissolved in IPA/2-butanol (10 vol.%) at 5 gL^{-1} concentration. Photo Credit: Guohua Hu, University of Cambridge and The Chinese University of Hong Kong.

Section S5. Spray coating of the formulated inks

The formulated inks have a viscosity of 2.2 mPas, indicating that the inks can be used with low viscosity dispersion deposition techniques, for instance, spray coating and inkjet printing (*4*, *32*, *55*). Note as discussed, the surface tension of the inks (~28 mNm^{-1}) ensures that the inks wet common substrates including such as Si/SiO_2, glass and PET for a consistent deposit.

Fig. S7a presents a sprayed 2H-MoS_2 film. In this case, 0.5 mL 2H-MoS_2 ink is sprayed onto PET at room temperature. SEM characterization of the sprayed 2H-MoS_2 film further demonstrates even flake distributions without discernible pinholes; Fig. S7b,c. Note that the sprayed 2H-MoS_2 PET film is sputtered with 6 nm thick gold for SEM characterization. We also measure spatial optical absorbance of the sprayed 2H-MoS_2 film at 550 nm with 1 cm step interval using UV-Vis spectroscopy. The acquired spatial absorbance (after subtraction of substrate absorbance) is 0.0448 ± 0.0019; Fig. S7d. This small standard deviation, 4.2%, in spatial absorbance demonstrates that the inks allow highly spatially consistent large-area coating. Fig. S7e presents the absorbance of 2H-MoS_2 films sprayed with varied ink volumes. The absorbance linearly scales with the ink volume: 0.0899 per mL, with a 2.1% standard deviation. This shows that the inks allow a good controllability over the optical densities of the deposited films.

The inks with insufficient stability for long-term inkjet printing can be suitably deposited with spray coating. Besides 2H-MoS_2, we also show in Fig. S7a sprayed films of graphene and In_2Se_3 (exfoliated via LPE). As shown, the sprayed films are visually uniform. Indeed, the respective measured spatial optical absorbance is 0.31533 ± 0.002 and 0.47256 ± 0.002, demonstrating standard deviations <1%. 5 mL inks are sprayed.

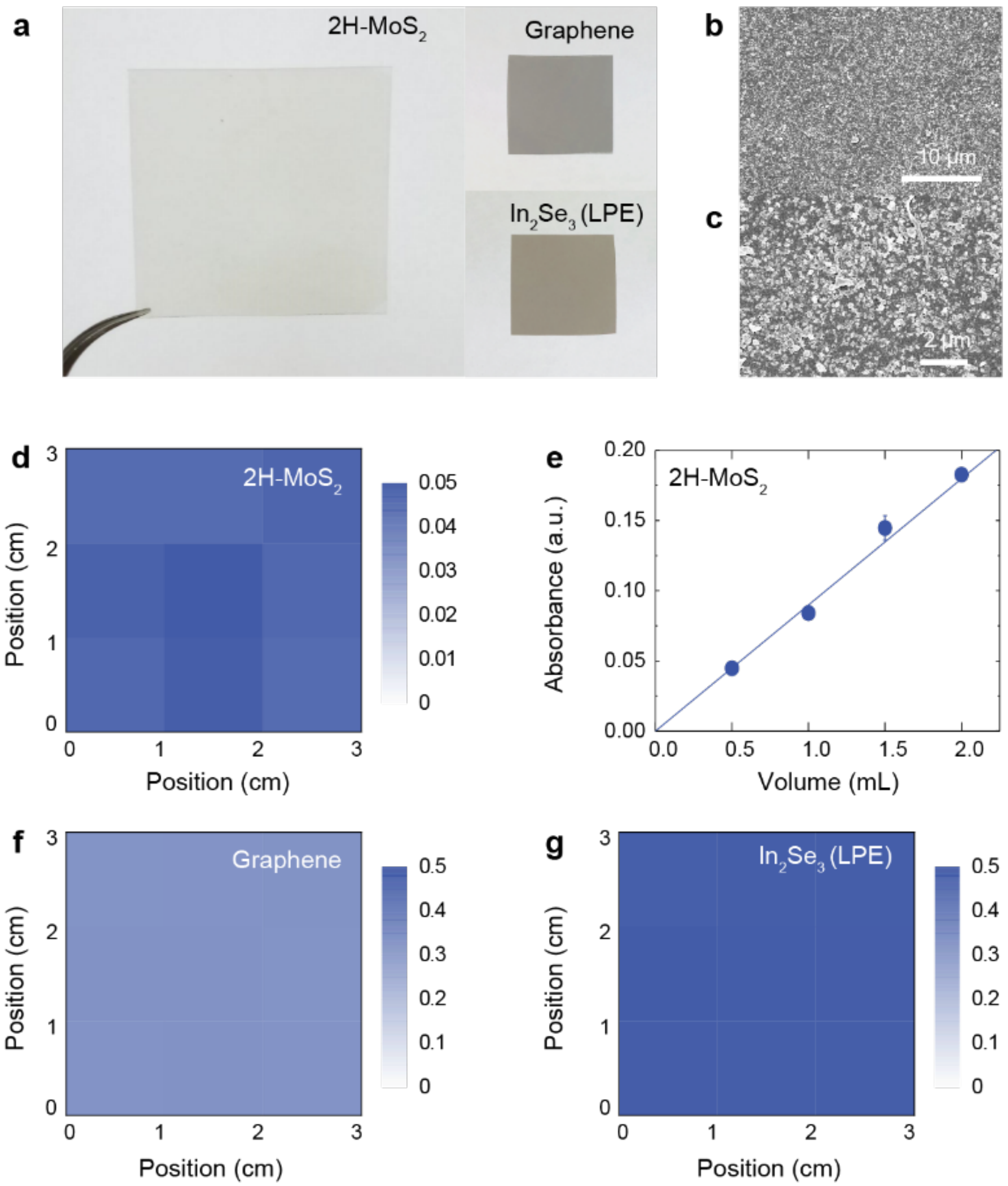

Fig. S7. Spray coating with the formulated inks in this work. (a) Photographs of sprayed films of 2H-MoS_2, graphene and In_2Se_3 (LPE) on PET. The films are ~3 cm × 3 cm. (b,c) SEM micrographs of the sprayed 2H-MoS_2 film. (d,f,g) Spatial absorbance at 550 nm of the sprayed films over a 3 cm × 3 cm region, step - 1 cm. (e) Optical absorbance at 550 nm of sprayed 2H-MoS_2 films as a function of the deposited ink volume (with absorbance from PET subtracted). Photo Credit: Guohua Hu, University of Cambridge and The Chinese University of Hong Kong.

Section S6. Optimization of printing parameters

As shown in Table S1, the formulated inks are characterized with a typical surface tension of ~28 mNm^{-1}, viscosity of ~2 mPas and density of ~0.8 gcm^{-3}, allowing the determination of a typical Z value of ~10, well within the optimal value range 1-14 for stable jetting. Indeed, during inkjet printing, we can observe stable jetting of single droplets from the printer stroboscopic camera (demonstrated with the 2H-MoS_2 ink); Fig. S8a. We control the dried droplet radius *r*' with varied substrate temperatures; Fig. S8b. As discussed, ideally the impingement of a droplet onto a pre-defined pattern should cause neither overspreading nor insufficient merging with neighboring droplets (*56*). Assuming a track of ink droplets (volume *V*') is printed in such an ideal case, the droplets then merge and form a line with uniform edges. Fig. S8c schematically shows a droplet impinging to this line with spacing of *d*'. Before solvent evaporation, the droplet has a spherical-cap geometry with radius of *r*', while the line has a cylindrical-cap geometry with radius of *R*'. The spherical-cap can be defined as:

$$V' = \frac{4}{3}\pi r'^3 \delta \tag{2}$$

where δ is the volume correction factor. Note that δ is expected to be very small when the droplet wets the substrate well, i.e. forms a small contact angle. On the other hand, δ approaches 1 for contact angles close to 180°. If *d*' is optimal, the impingement is then ideal such that the droplet extends the line by *d*' while the radius of the line remains *R*'. The cylindrical-cap is then defined as:

$$\frac{V'}{d'} = \pi R'^2 \delta' \tag{3}$$

where δ' is the volume correction factor for cylindrical-cap. Therefore, the relationship between *R*' and *r*' is:

$$\frac{R'}{r'} = \sqrt{\frac{4\delta}{3d'/r'\delta'}} \tag{4}$$

In addition, as shown in Fig. S8c, the impinging distance from the droplet to the end of the line (defined herein as the 'bead') is:

$$D' = d' - R' \tag{5}$$

Therefore, the relationship between *D*' and *r*' is:

$$\frac{D'}{r'} = \frac{d'}{r'} - \sqrt{\frac{4\delta}{3d'/r'\delta'}} \tag{6}$$

The morphologies of the printed patterns are governed by the droplet impinging behavior, which is essentially defined by the above parameters. As depicted in Fig. S8d, when *d*' is excessively small (*d*' < *R*', i.e. *D*' < 0 from equation 5), the droplet lands on the bead of the line and expands around the bead rather than creating its own contact line, forming 'stacked coins' or 'bulged' line morphologies. However, when *d*' is large (2*R*' < *d*' < 2*r*', i.e. *D*' > *R*' from equation 5), the impinging of the droplet with the bead is restrained, forming 'scalloped' lines. For excessively large *d*' (*d*' > 2*r*'), the droplet does not impinge onto the line, forming 'isolated droplets'. For *d*' values between the bulged and scalloped scenarios (i.e. 0 < *D*' < *R*'), the droplet impinges onto the bead and forms contact line with uniform edges.

Fig. S8f,g present respective printed lines of 2H-MoS_2 on Si/SiO_2 with single print repetition with variable *d*' and varied *r*'. Indeed, as shown, varying *d*' and *r*' for the 2H-MoS_2 ink produces vastly different morphologies, varying from stacked coins, bulged lines, uniform lines, scalloped lines and eventually to isolated droplets. For the *r*' ~34 μm case in Fig. S8f, the line with uniform edges begins to emerge when *d*' is 35 μm, and the line width is ~76 μm (i.e. *R*' is ~38 μm). Using the empirically obtained parameters above (*r*' ~34 μm, *R*' ~38 μm, *d*' = 35 μm) and *V*' of 10 pL (defined by the ink cartridge used in this work), the correction factors δ and δ' in equation 2 and equation 3 are determined, giving δ/δ' as 0.96. The normalized *R*' and *D*' values as a function of normalized *d*' (equation 4 and equation 6, respectively) are therefore plotted in Fig. S8e. Considering the above boundary condition for uniform line edges, 0 < *D*' < *R*', this suggests that the printed morphology with uniform line edges can be found when *d*'/*r*' is ~1.1-1.7. We note that similar studies of such droplet impingement behavior have been previously carried out with metal nanoparticle based inks (*56*).

The peak-to-peak edge roughness (Δ*d*') along the printed lines is a key consideration for the morphology of printed patterns. Here, we define the edge roughness as $(L_a - L_b)/2$, where L_a and L_b are the maximum and minimum width of a printed line, respectively. Fig. S8h replots the bulged line defined at 25 μm, where the maximal width L_1 is ~119 μm and the minimal width L_2 is ~108 μm. The roughness therefore is calculated as $(L_1 - L_2)/2$ = (119 – 108)/2 μm = 5.5 μm. For the scalloped line defined at 55 μm in Fig. S8i, L_3 and L_4 are ~78 μm and ~62 μm, respectively. The roughness is calculated as $(L_3 - L_4)$ 2 = (78 – 62)/2 μm = 8 μm.

Having defined the edge roughness, we also confirm the above optimal printing parameters experimentally. Fig. S9a presents a contour plot of the edge roughness with respect to *d'* and *r'*. We consider Δ*d*' <2 μm as the criterion for uniform line edges. Fig. S9a shows that the 'uniform edges' region has distinct boundaries to the bulging and scalloping regimes, suggesting that the optimum *d*'/*r*' ratio obtained empirically is ~1.0-1.6, correlated with the above prediction and other ink systems such as polymer based and metal nanoparticle based inks (*56*, *57*). This therefore suggests a general printing guidance for our formulated ink: the droplet spacing should be set as ~1.0-1.6 the deposited droplet radius for optimal printed morphologies. We note that the stacked coins region can also be exploited to print lines with uniform edges, but with larger width of the printed lines.

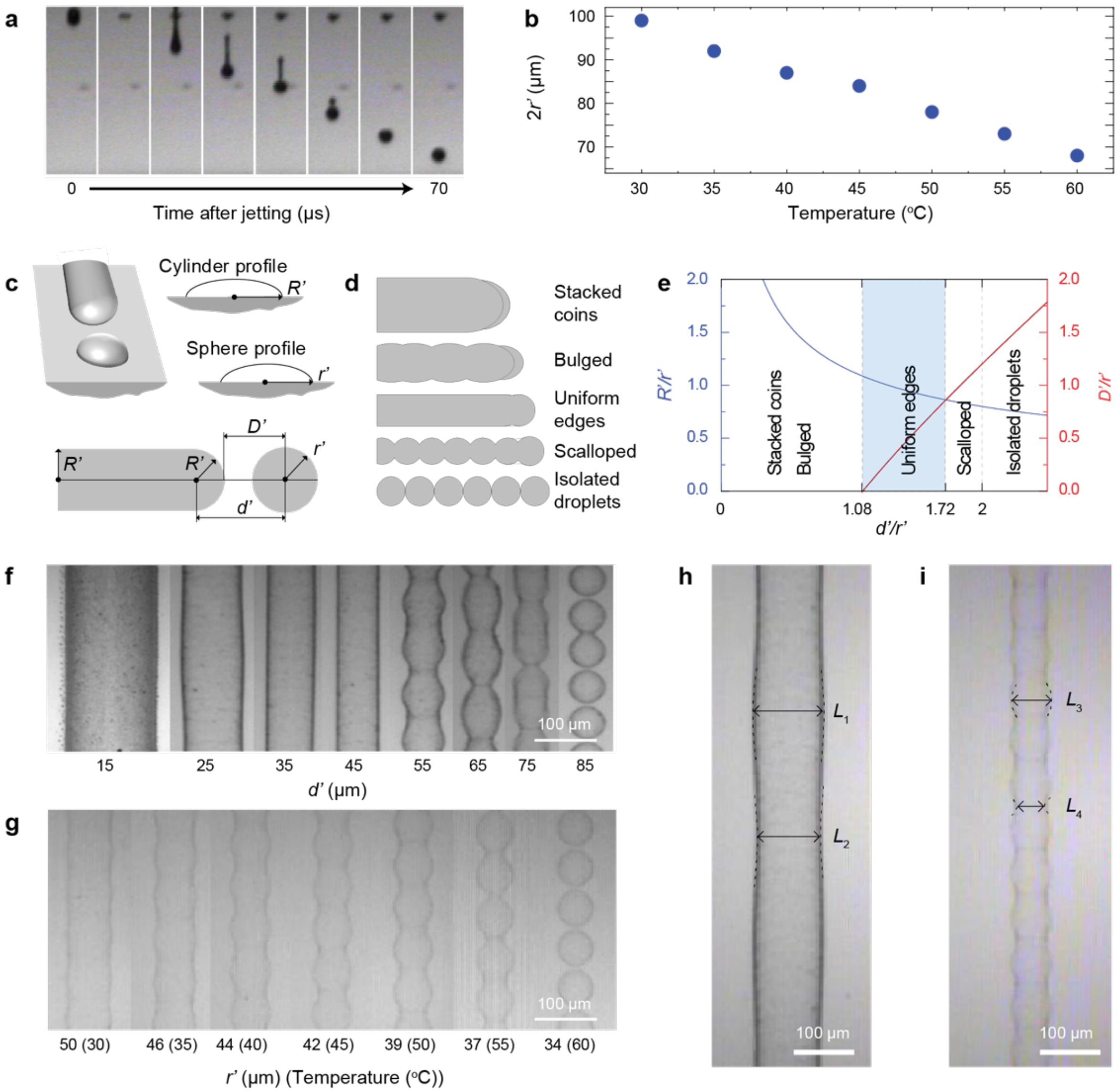

Fig. S8. Inkjet printing with the formulated inks in this work. (a) Ink droplet jetting sequence observed through the printer stroboscopic camera. (b) Radius (*r*') of dried 2H-MoS_2 ink droplets on Si/SiO_2 with respect to the substrate temperature. (c) Schematic figure with top view showing a droplet of a radius of *r* deposited to a defined cylindrical-cap line of a radius of *R*' with a droplet spacing of *d*'. (d) Schematic figures showing printed morphologies defined at varied droplet spacings. (e) Dimensionless *R*' versus *d*' plot predicting that printed morphology with uniform line edges is achieved when *d*' is 1.1-1.7 of *r*'. Photographs of typical printed lines with single print repetition under (f) varied *d*', *r*' is 34 µm, and (g) varied *r*', *d*' is 85 µm. Printed lines on Si/SiO_2 with *d*' of (h) 25 µm and (i) 55 µm, with 34 µm *r*'.

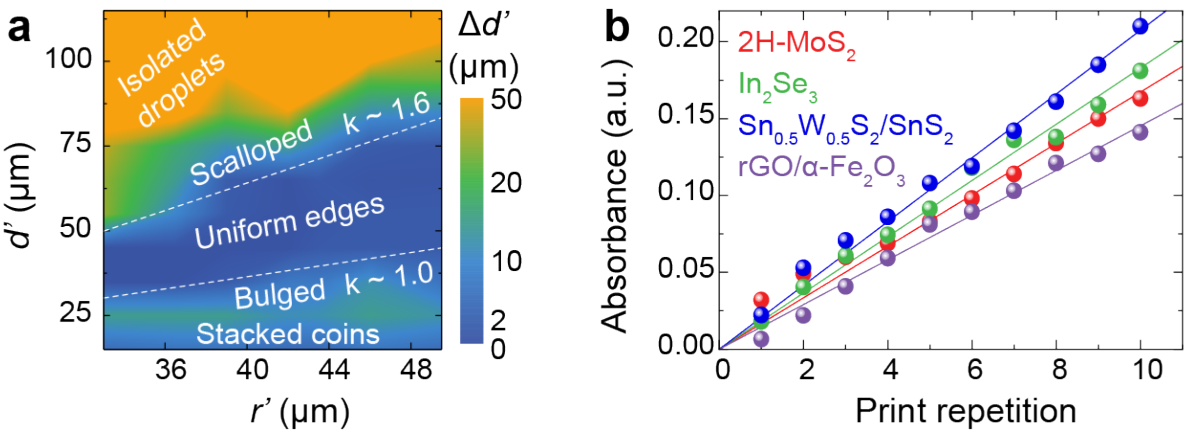

Fig. S9. Edge roughness of printed lines and optical absorbance of printed patterns. (a) Measured line edge peak-to-peak roughness Δ*d*' of the inkjet-printed 2H-MoS_2 lines on Si/SiO_2 with respect to *d*' and r', showing uniform edges with Δ*d*' < 2 μm are defined with *d*'/*r*' ~1.0-1.6. (b) Under the optimal printing parameters, the measured optical absorbance of some types of printed 2d crystals on glass with respect to print repetition at 550 nm, showing slope errors of 2.5%, 1.6%, 1.4% and 1.9%. Glass absorbance is subtracted.

Section S7. Inkjet-printed nonlinear optical devices

As presented in the manuscript, we first focus on small-scale fabrication of 2d crystal-based nonlinear optical devices, which are critical components for the development of ultrafast lasers. Such ultrafast laser technology continues to have a major impact in, for example, the observation of fast processes in nature as well as in industrial laser manufacturing and biomedical imaging (*58*). The high nonlinear susceptibilities and ultrafast carrier dynamics of a wide variety of 2d crystals, including those of 2H-MoS_2, are promising for the development of fast nonlinear optical switches known as saturable absorbers (SAs), which can be inserted into laser cavities to convert a low-power continuous-wave output into a train of high-peak-power ultrashort pulses (*22*). The properties of these ultrashort pulses are affected by the characteristics of the SA and hence it is of primary importance to have high device-to-device consistency. The demonstrated scalable patterning capability of our inks, in particular uniform flake distribution and precise control over the optical density, is ideal to deliver printed 2d crystal based SA fabrication.

Fig. S10a presents a photograph of an array of inkjet-printed 2H-MoS_2 SAs, highlighting high spatial consistency and uniformity. For printing of the SAs, the 1.5 μm thick PET is used directly as provided without any surface treatment. For ease of handing, this PET is laminated onto a 100 μm thick PET before printing; it can be easily peeled off for device integration after printing. Spatial color intensity (I_c) is then extracted for detailed investigation (Fig. S10b). To do this, we convert this photograph into a data worksheet of color intensity using OriginLab with the 'Matrix: Covert to Worksheet' function. The extracted data of the background is normalized to 0, whereas the maximum spike data-point across the SA region is normalized to 1. This spatial color intensity mapping shows that the normalized I_c is consistent within each individual SA and without discernible variations across the investigated SAs. The average I_c across the SA array is 0.811 ± 0.023, giving an area-to-area standard deviation of ~2.8%. We then characterize the nonlinear optical properties of the inkjet-printed 2H-MoS_2 SAs using an open-aperture Z-scan set-up (Fig. S10c). The acquired Z-scan datasets are well fitted with a two-level saturation model: $\alpha(I) = (\alpha_l - \alpha_{ns})/(1 + I/I_{sat}) + \alpha_{ns}$ (*59*, *60*), where α_l is the linear absorption at low intensity and α_{ns} is the nonsaturable absorption at high intensity, I is the instantaneous incident intensity, and I_{sat} is the saturation intensity. The modulation depth (α_m) of a device is given by: $\alpha_m = \alpha_l - \alpha_{ns}$. Consequently, I_{sat} can be defined as the intensity required to reduce the absorption $\alpha(I)$ to $\alpha_l - (\alpha_m/2)$. The acquired spatial optical properties are highly consistent, with linear absorption (α_l) of 6.9 ± 0.13%, non-saturable absorption (α_{ns}) of 4.5 ± 0.22%, modulation depth (α_m) of 2.38 ± 0.11%, and saturation intensity (I_{sat}) of 3.37 ± 0.11 $MWcm^{-2}$. The properties of inkjet-printed SAs of WS_2 and $MoSe_2$ are shown in Fig. S11a,b. The spatial saturation intensities are 2.26 ± 0.14 $MWcm^{-2}$ and 3.27 ± 0.13 $MWcm^{-2}$ for WS_2 and $MoSe_2$, respectively. The spatial optical linear absorptions are 8.4 ± 0.10% and 8.2 ± 0.10%, respectively. The spatial optical non-saturable absorptions are 6.6 ± 0.22% and 5.3 ± 0.17%, respectively. The spatial optical modulation depths are 1.59 ± 0.20% and 2.9 ± 0.16%, respectively. This demonstrates that the inkjet-printed TMD SAs are highly uniform in the linear and nonlinear optical properties, with <5% standard deviation, suggesting that all the functional properties of the inkjet-printed SAs are spatially consistent.

The schematic erbium-doped (Er-doped) fiber laser set-up as in Fig. 4a is replotted with detail in Fig. S10d. The laser cavity consists of a single-clad Er-doped active fiber (YOFC EDF7/6/125-23, 4.8 m length, 17.8 ps^2km^{-1} group velocity dispersion (GVD)), co-pumped by a 980 nm laser diode through a 980/1550 wavelength division multiplexer (WDM), a polarization independent isolator (ISO) to ensure unidirectional propagation, a polarization controller (PC) to enable a continuous

adjustment of the net cavity birefringence and a 20:80 fused fiber output coupler (OC) for both spectral and temporal diagnostics. The ISO, PC and OC are fiber-pigtailed with single-mode fiber (SMF) with a GVD of -22 ps^2km^{-1}, while the WDM comprises single-mode HI1060 with a GVD of -7 ps^2km^{-1}. The entire cavity length is 10.18 m with a net cavity dispersion of 0.078 ps^2, which permits the laser to operate in the dispersion-managed soliton regime (*61*, *62*).

When in operation, the SAs allow stable, self-starting mode-locked high-peak-power femtosecond pulses to be generated. Fig. S10e presents a typical autocorrelation trace of the generated pulses. We then measure the laser operation stability of the 16 randomly selected 2H-MoS_2 SAs from this array. The SA devices are integrated into the laser using a free-space configuration, such that we can change the sample without mechanically perturbing the cavity fiber which could induce change in the laser operation stability by varying the birefringence. This is achieved with an imaging system using a pair of 10 mm focal length lenses, where the SA devices are held at the focal point on a precision translation stage. Light transmitted through the device is collected by a second pair of 10 mm lenses and coupled back into fiber. Assessment of 16 individual SAs shows uniform pulse duration (τ) distribution, with 68.8% SAs within 1σ (3.3% spread from the μ value) and 100% finished device yield (Fig. 4c). We argue that the spread of τ is within acceptable statistical process variations for scalable manufacturing of ultrafast lasers, addressing a major limitation of solution-processed 2d crystal based nonlinear optical devices in the past decade (*22*, *63*).

Besides the discussion in the manuscript, the measured generated ultrashort pulses from the 2H-MoS_2 SAs are presented in Fig. S10e,f. The corresponding spectral profiles also exhibit a high uniformity in laser operation: the position of the fundamental radio frequency spectra is 20.1755 ± 0.0016 MHz (0.0079% standard deviation) (Fig. S10g); the pulses are centered at 1558.46 ± 0.44 nm (0.03% standard deviation), with a full width at half maximum of 28.11 ± 0.39 nm (1.4% standard deviation) (Fig. S10h,i).

The inkjet-printed SA devices of WS_2 and $MoSe_2$ are also integrated into the Er-doped ultrafast fiber laser cavity for ultrashort laser pulse generation. Typical respective output pulse properties are shown in Fig. S11c,d. By incorporating WS_2 and $MoSe_2$ SAs into the laser cavity, the measured spectral FWHM is 27.59 nm and 27.85 nm, centered at 1558.66 nm and 1558.42 nm, respectively. The deconvolved pulse durations are 166.43 fs and 163.67 fs, respectively. The radio frequency spectra also show high signal-to-noise ratios, exceeding 60 dB.

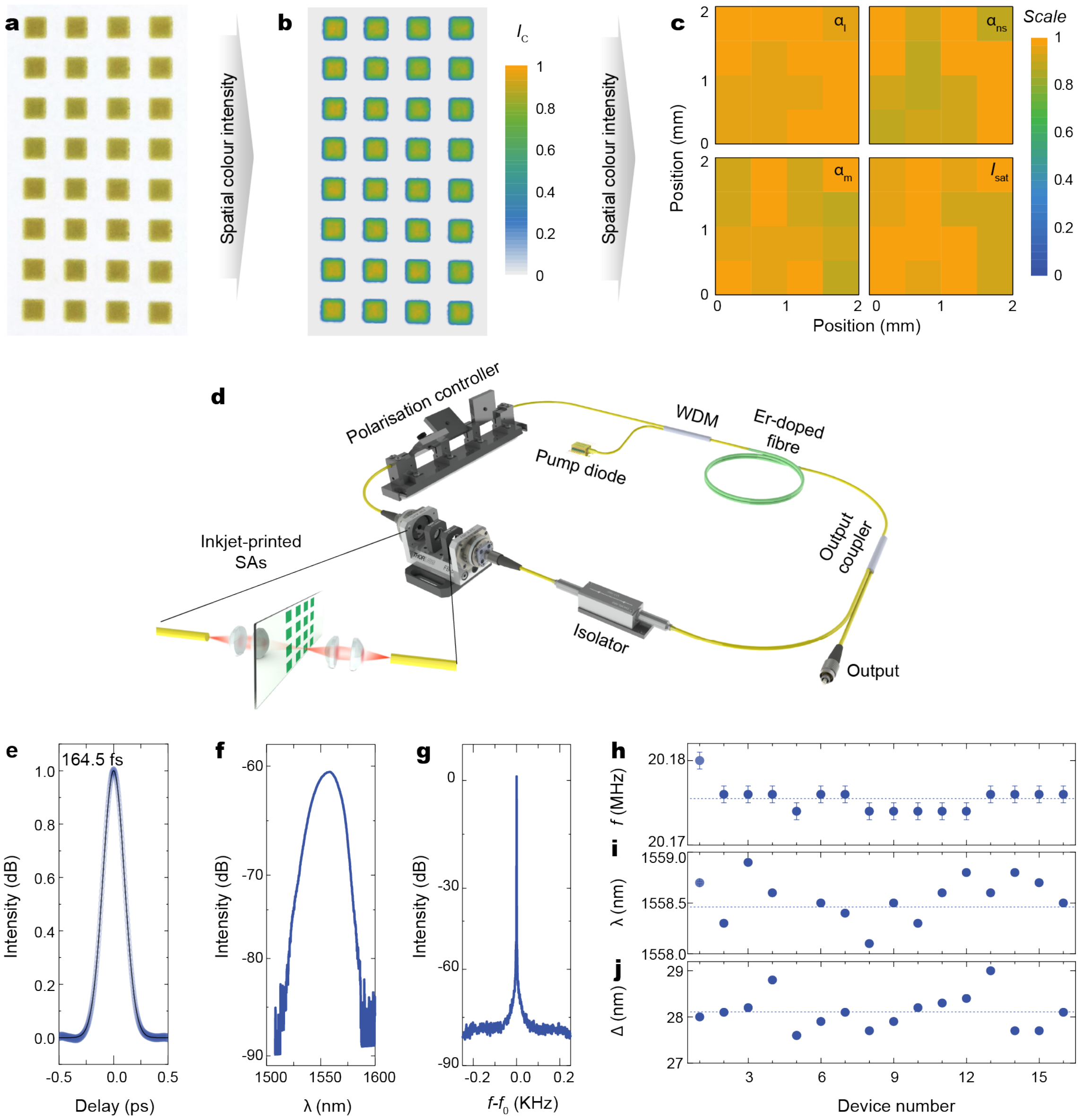

Fig. S10. Inkjet-printed MoS_2 nonlinear optical devices. (a) Photograph of inkjet-printed 4 × 8 2H-MoS_2 SA array on ultrathin PET (thickness 1.5 µm, laminated onto 100 µm thick PET), the background is paper, and (b) its spatial color intensity (I_C). The dimension of individual SAs is 2 mm × 2 mm, the color intensity of the background is normalized to 0 and the intensity of the maximum spike is normalized to 1; (c) Normalized spatial optical linear absorption (α_l, %), non-saturable absorption (α_{ns}, %), modulation depth (α_m, %) and saturation intensity (I_{sat}, $MWcm^{-2}$) of typical 2H-MoS_2 SAs, data acquired by Z-scan at 1560 nm, spatial step - 0.5 mm. (d) Schematic figure of the Er-doped ultrafast fiber laser cavity. (e) Autocorrelation trace of typical generated ultrashort pulses fitted with a Gaussian curve. (f) Typical output laser pulse spectra and (g) radio frequency spectra at the cavity fundamental repetition frequency (f_0; 20.176 MHz) of the 2H-MoS_2 SAs. (h) Measured position of the fundamental radio frequency spectra, (i) measured spectral central wavelength (λ), and (j) full width at half maximum (Δ) of the ultrashort pulses generated from 16 individual 2H-MoS_2 SA devices. The dashed lines indicate the average values, and the error bars indicate the errors from the measurement system.

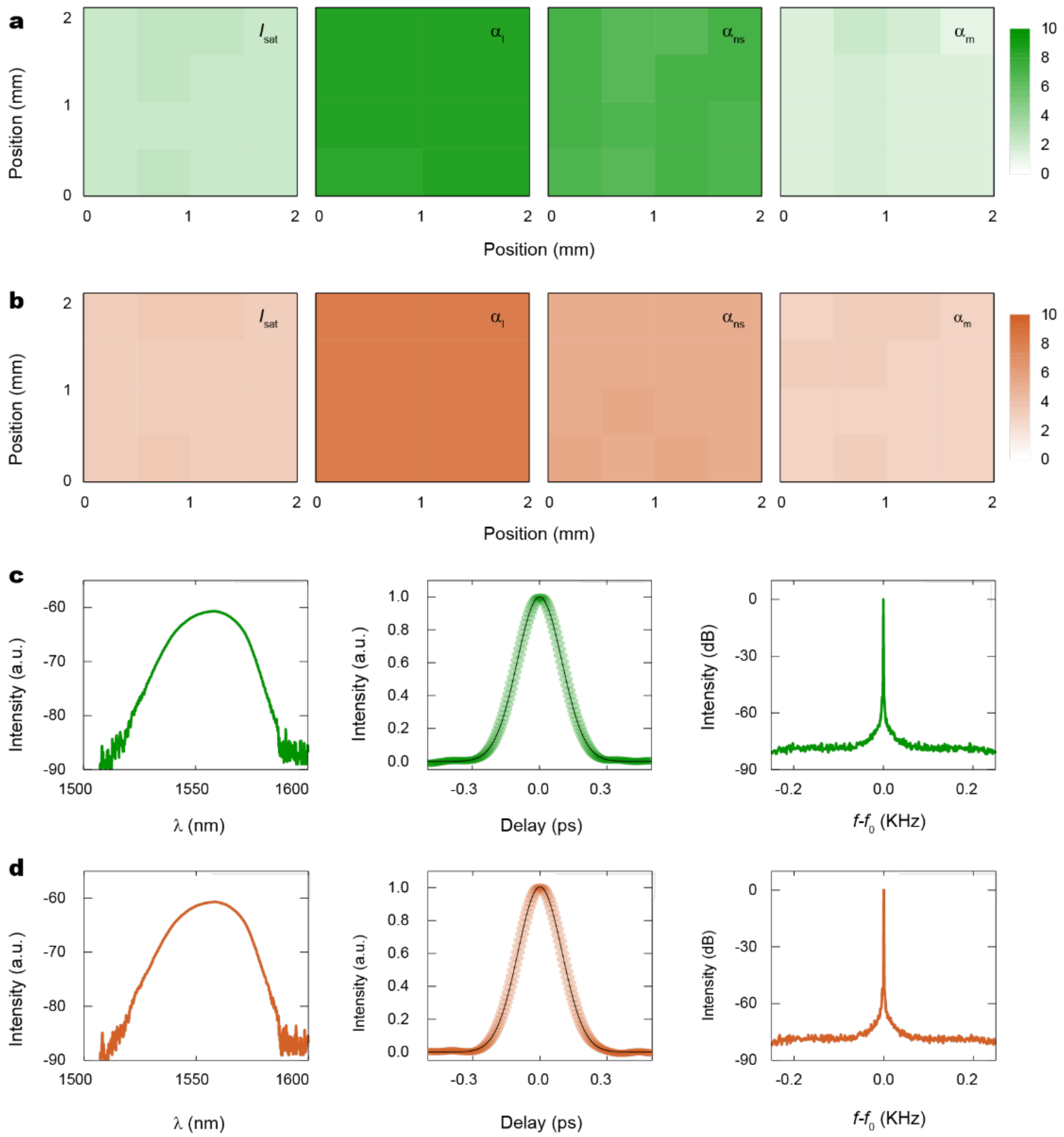

Fig. S11. Inkjet-printed WS_2 and $MoSe_2$ nonlinear optical devices. Spatial saturation intensity (I_{sat}, $MWcm^{-2}$), optical linear absorption (α_l, %), optical non-saturable absorption (α_{ns}, %), and optical modulation depth (α_m, %) at 1560 nm of typical inkjet-printed SAs of (a) WS_2 and (b) $MoSe_2$ over a 2 mm × 2 mm region, spatial step - 0.5 mm. Ultrafast laser operation of the inkjet-printed (c) WS_2 and (d) $MoSe_2$ SAs: output laser pulse spectra, autocorrelation trace of output pulses fitted with a Gaussian curve, and radio frequency spectra at 20.176 MHz.

Section S8. Inkjet-printed gas sensors

We also investigate large-scale manufacturing of gas sensors. Sensors find widespread uses in environmental monitoring, industrial processes, agriculture, smart buildings and increasingly, healthcare. As a material platform, rGO/α-Fe_2O_3 offers high sensitivity towards NO_2 at room-temperature and hence is promising for such sensor developments (*23*). More importantly, as demonstrated, rGO/α-Fe_2O_3 can be formulated into inkjet printable ink and deliver highly uniform and consistent deposition through our formulation. We believe this represents an important avenue towards high sensitivity, simple and cost-effective sensor fabrication of rGO/α-Fe_2O_3 and similar functional materials.

Fig. 4e in the manuscript shows an array of 50 fully-inkjet-printed rGO/α-Fe_2O_3 sensors. In these sensors, the inkjet-printed rGO/α-Fe_2O_3 onto interdigitated silver electrodes (Ag IDEs) acts as the active sensing layer. We use PET with a porous coating that is specifically designed for silver printing, to avoid short-circuits of the Ag IDEs that are otherwise seen with PET substrates (Fig. S12a,b). Optical microscopy shows highly uniform deposit of rGO/α-Fe_2O_3 over the Ag IDEs; Fig. 4e. When exposed to NO_2, the printed sensors show a strong response, even at concentrations down to 200 ppb. The average (maximum) responsivity (R_{gas} - the change in the device resistance) is ~24% (~37%) over the investigated concentration range (200-1,000 ppb, in steps of 200 ppb); Fig. S12c. We then assess the device-to-device consistency in R_{gas}. For this, we measure the maximum R_{gas} at 1 ppm NO_2 of this sensor array (3 out of the 50 devices are short-circuited during electrode fabrication using inkjet printing of silver inks), and find that 78.7% sensors are within ± 1σ (2.5% spread from their μ value); Fig. S12c, Fig. 4g. Note that the baseline drift, common for chemiresistive sensors, can be addressed by saturating the sensors in NO_2; Fig. S12d. This demonstration reinforces the fact that our formulation enables scalable device manufacturing through inkjet printing with high device-to-device consistency.

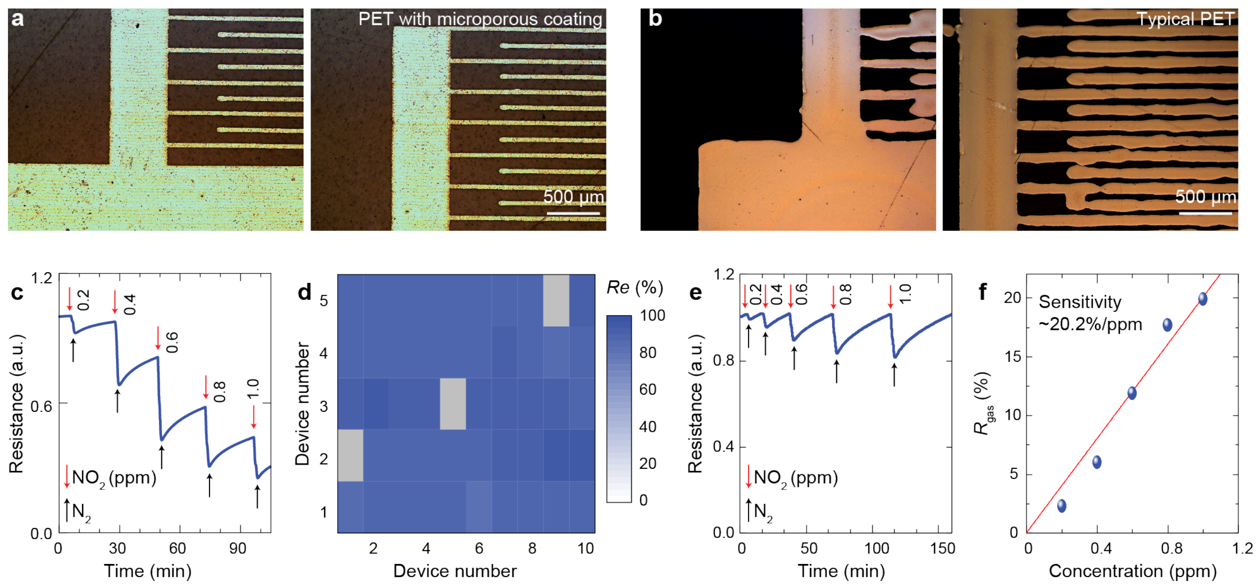

Fig. S12. Inkjet-printed rGO/α-Fe_2O_3 gas sensors. Optical micrographs of inkjet-printed commercial silver (Sigma) interdigitated electrodes at different regions on (a) the Mitsubishi silver printing PET substrate with porous coating, and (b) regular PET substrate. (c) Typical measured response under exposure to NO_2 in the initial measurement cycles. (d) Mapping of the measured responsivities (R_{gas} – the change in the device resistance) to 1 ppm NO_2 of the all-inkjet-printed rGO/α-Fe_2O_3 sensor array shown in Fig. 4d in the manuscript. The 'null' grey spots represent short-circuited devices. (e) Typical measured stabilized response under exposure to 1 ppm NO_2 for 30 min. (f) The measured stabilized R_{gas} as a function of NO_2 concentration, giving a stabilized sensitivity of 20.2% per ppm to NO_2.

Section S9. Inkjet-printed photodetectors

Semiconducting TMDs are an interesting material platform for the photodetector development due to their transition bandgap spanning the visible to near-infrared (NIR) wavelength region (*1*). Towards cost-effective real-world device manufacturing, inkjet printing of TMDs has been successfully exploited in laboratorial photodetector demonstrations (*6*, *64*, *65*). However, challenges exist. The scalability is significantly hindered (≤20 devices) due to the limitations in the solution-processed 2d crystal dispersions, and as such a rigorous characterization of device-to-device variations was not been performed. The devices, based on solution-processed TMDs, also show pretty poor performances compared to the ones based on micro-mechanically cleaved and chemical vapor deposition-grown materials. Practical applications therefore require further substantially improved ink formulation and materials functionality. Nevertheless, with a highly controlled and uniform deposition capability, our ink formulation solves the challenge of scalable device fabrication, meeting the needs for wafer-scale manufacturing.

Towards this goal, we demonstrate 4,500 (100 × 45) 2H-MoS_2 photodetectors by inkjet printing 2H-MoS_2 onto interdigitated gold electrodes on a single silicon wafer; Fig. 4h. As shown, the printed 2H-MoS_2 array is visually highly identical and consistent; Fig. 4i. Fig. 4j,k are zoomed-in optical and false-colored SEM images of a single device and the printed 2H-MoS_2, respectively, showing an even flake distribution over the electrodes. To assess the device-to-device consistency of the wafer-scale photodetectors shown in Fig. 4h in the manuscript, we stochastically measure the electrical conductance (G) of 165 individual devices across the entire array (Fig. S13a). Our measurements confirm that 61.2% (101 out of 165) of the devices have G values within ± 1σ (11.3% spread from the μ value), with 161 out of 165 (97.6%) within the 2σ range (Fig. S13b), again highlighting high device-to-device consistency and manufacturability using our ink formulation. As opposed to this, an array of 5 × 10 2H-MoS_2 photoreactors fabricated using the solution-processed NMP based dispersion (discussed in Fig. 1a) shows uncontrollable, varied print patterns of MoS_2 with uneven flake distribution over the electrodes; Fig. S14a,b. This leads to a significantly wider spread in the G values across the device array; Fig. S14c. Gaussian fitting gives a 29.1% spread for 1σ from the μ value; Fig. S14d.

We then characterize the photoresponse of the printed photodetectors. As shown in Fig. S13c, compared to the dark current, the current under excitation is typically over one order of magnitude larger, with responsivity (R_{photo}) of up to 150 μAW^{-1}. Fig. S13c presents the typical photoresponse with respect to time, showing that the current under excitation remains stable, with <5% error. After confirming the stable device operation, as discussed, we then investigate the device-to-device consistency in terms of R_{photo}. As presented in Fig. S13d and Fig. 4i, R_{photo} from a randomly-selected 5 × 10 device array is highly consistent (6 out of the 50 devices are short-circuited during electrode fabrication using lithography), with 68.2% of the devices within ± 1σ (9.1% spread from the μ value) range, and 97.7% within ± 2σ which we believe is sufficient for industrial-scale manufacturing. The above demonstrations envisage the prospect of our ink formulation of 2d crystals, their heterostructures and hybrids in real-world wafer-scale manufacturing with highly consistent device-to-device performance.

Fig. S15a,c present the mapping of measured R_{photo} values under 40 μW excitation at 5 V for WS_2 and $MoSe_2$ photodetector arrays (50 devices), respectively. As shown, the measured R_{photo} values for the WS_2 photodetectors are highly consistent. Their measured R_{photo} values can also be well-fitted with Gaussian distribution, with fitted μ and σ of 2.6 μAW^{-1} and 0.3 μAW^{-1}, respectively; Fig. S15b. Therefore, 100% of the measured R_{photo} values are distributed within ± 3σ. The

measured R_{photo} values for the $MoSe_2$ photodetectors, however, show large variations. Fig. S15d presents typical time response of $MoSe_2$ photodetector, showing that the current decreases upon light excitation. We propose that the large R_{photo} variations of the $MoSe_2$ photodetector array are due to the instability of $MoSe_2$ but not the device fabrication strategy. Nevertheless, the R_{photo} of both the 2H-MoS_2 and WS_2 photodetector arrays exhibit high device-to-device uniformities.

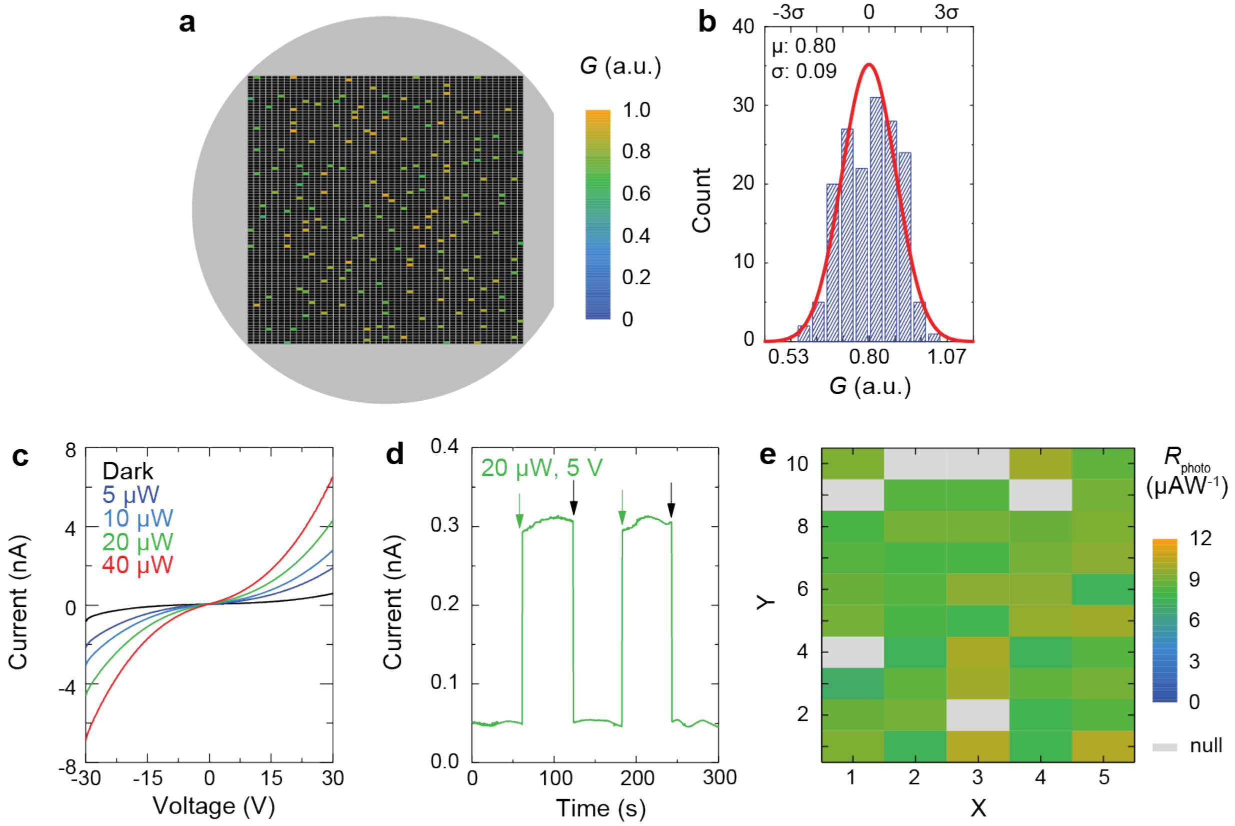

Fig. S13. Inkjet-printed wafer-scale MoS_2 photodetectors. (a) Normalized electrical conductance (G) map of the wafer-scale photodetectors shown in Fig. 4h in the manuscript, the spots indicate the measured devices. (b) Gaussian fitting of the measured *G*, with 101 out of 165 (61.2%) within ± 1σ, 161 out of 165 (97.6%) within ± 2σ, and 165 out of 165 (100%) within ± 3σ. (c) Typical device current response to varied excitation powers. (d) Typical time response, bias 5 V, the green and black arrows indicate excitation on and off. (e) Responsivity (R_{photo}) map of a 50 device array under 40 μW, bias 5 V, the 'null' grey spots represent short-circuited devices. A 635 nm excitation laser is used.

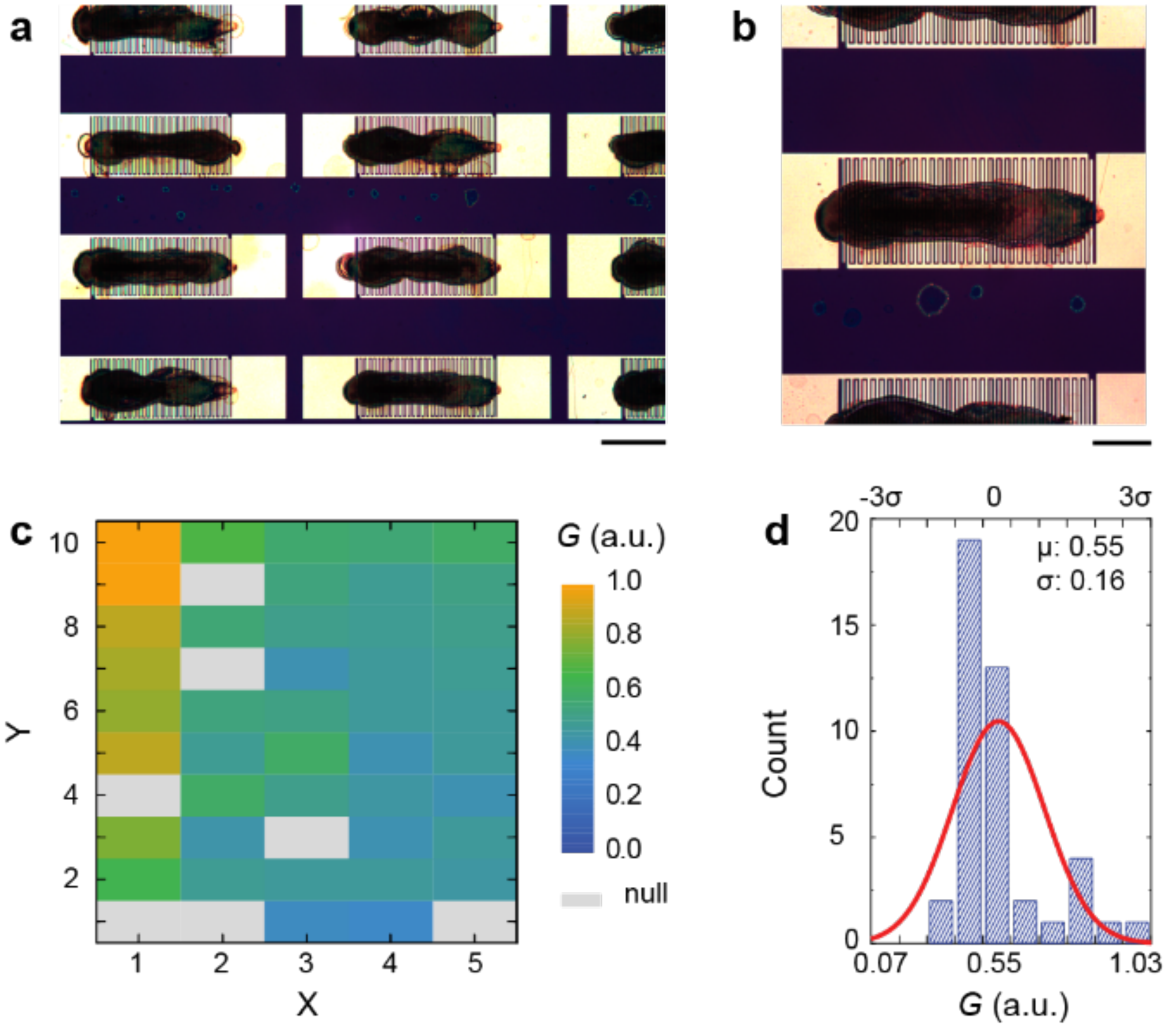

Fig. S14. Inkjet-printed MoS_2 photodetectors with the solution-processed NMP based dispersion. (a, b) Optical micrographs of 2H-MoS_2 photodetector devices fabricated using the as-produced NMP based dispersion discussed in Fig. 1a, showing uncontrollable, varied printed MoS_2 patterns with uneven flake distribution over the electrodes, respective scale bar 200 µm and 100 µm. (c) Normalized electrical conductance (G) map of the photodetector array printed with the NMP based dispersion, the 'null' grey spots represent short-circuited devices. (d) Gaussian fitting of the measured *G* values, showing a 29.1% spread from the µ value.

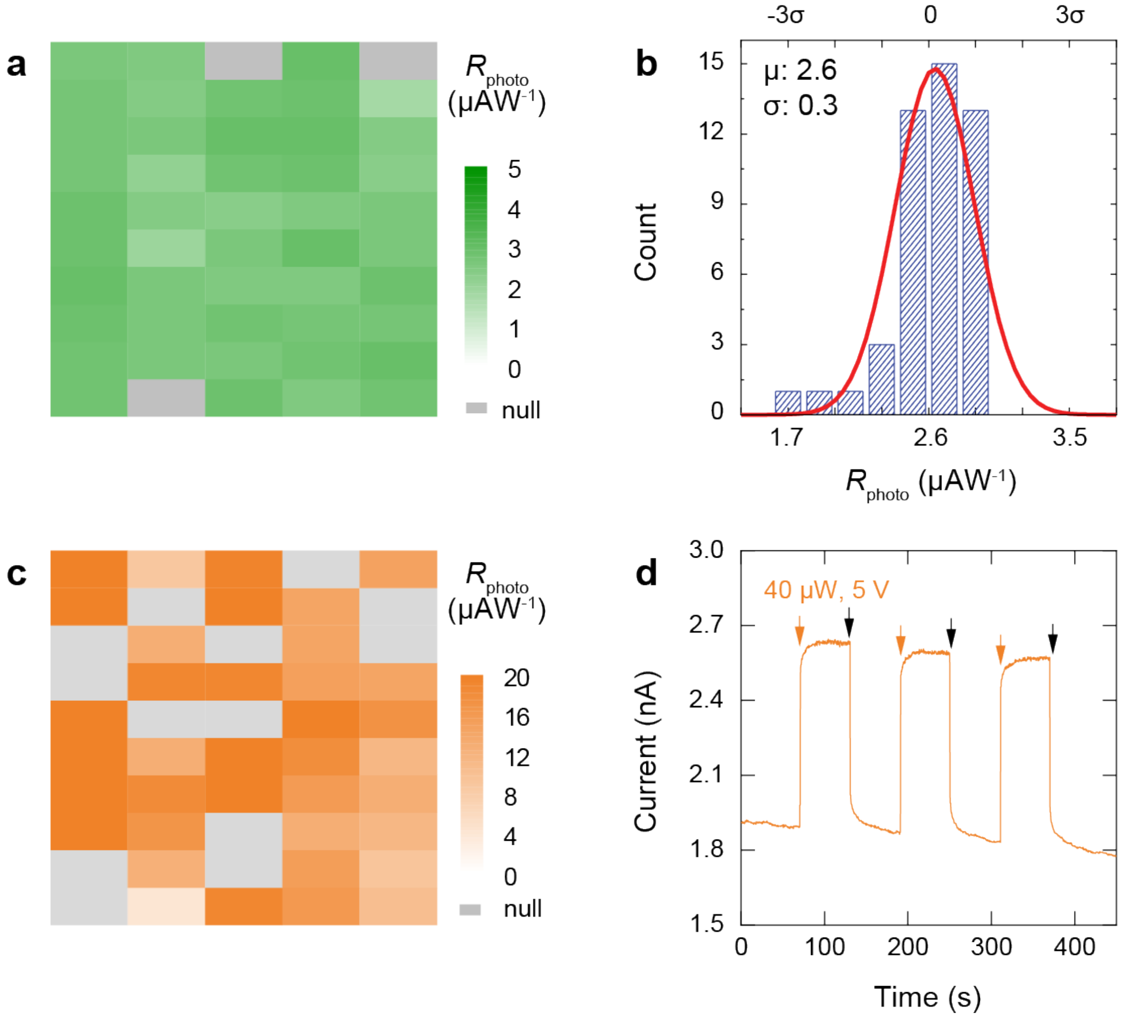

Fig. S15. Inkjet-printed WS_2 and $MoSe_2$ photodetectors. (a) Mapping of R_{photo} under 40 µW at 5 V for 50 WS_2 photodetector devices, and (b) the corresponding Gaussian fitting, with 37 out of 47 (78.7%) within ± 1σ, 45 out of 47 (95.7%) within ± 2σ, and 47 out of 47 (100%) within ± 3σ. 3 devices are short-circuited, represented by the 'null' grey spots. (c) Mapping of R_{photo} under 40 µW at 5 V for $MoSe_2$ photodetectors, and (d) the corresponding typical time response. The orange and black arrows indicate excitation on and off. The 'null' grey spots represent short-circuited devices. A 635 nm excitation laser is used.
